# Dislocation and strain mapping in metamorphic parabolic-graded InGaAs buffers on GaAs

**DOI:** 10.1007/s10853-023-08597-y

**Published:** 2023-06-07

**Authors:** Nicholas Stephen, Praveen Kumar, Agnieszka Gocalinska, Enrica Mura, Demie Kepaptsoglou, Quentin Ramasse, Emanuele Pelucchi, Miryam Arredondo

**Affiliations:** 1 School of Mathematics and Physics, Queen’s University Belfast, University Road, Belfast, UK; 2 Tyndall National Institute, University College Cork, “Lee Maltings”, Dyke Parade, Cork, Ireland; 3 Shared Instrumentation Facility, Colorado School of Mines, Golden, CO USA; 4 SuperSTEM Laboratory, SciTech Daresbury Campus, Daresbury, UK; 5 Department of Physics, University of York, Heslington, York UK; 6 School of Chemical and Process Engineering and School of Physics and Astronomy, University of Leeds, Leeds, UK

## Abstract

We investigate different architectures for parabolic-graded InGaAs metamorphic buffers grown on GaAs using transmission electron microscopy techniques. The different architectures include InGaP and AlInGaAs/InGaP superlattices with different GaAs substrate misorientations and the inclusion of a strain balancing layer. Our results correlate: (i) the density and distribution of dislocations in the metamorphic buffer and (ii) the strain in the next layer preceding the metamorphic buffer, which varies for each type of architecture. Our findings indicate that the dislocation density in the lower region of the metamorphic layer ranges between 10^8^ and 10^10^ cm^−2^, with AlInGaAs/InGaP superlattice samples exhibiting higher values compared to samples with InGaP films. We have identified two waves of dislocations, with threading dislocations typically located lower in the metamorphic buffer (~ 200–300 nm) in comparison to misfit dislocations. The measured localised strain values are in good agreement with theoretical predications. Overall, our results provide a systematic insight into the strain relaxation across different architectures, highlighting the various approaches that can be used to tailor strain in the active region of a metamorphic laser.

**Graphical abstract:**

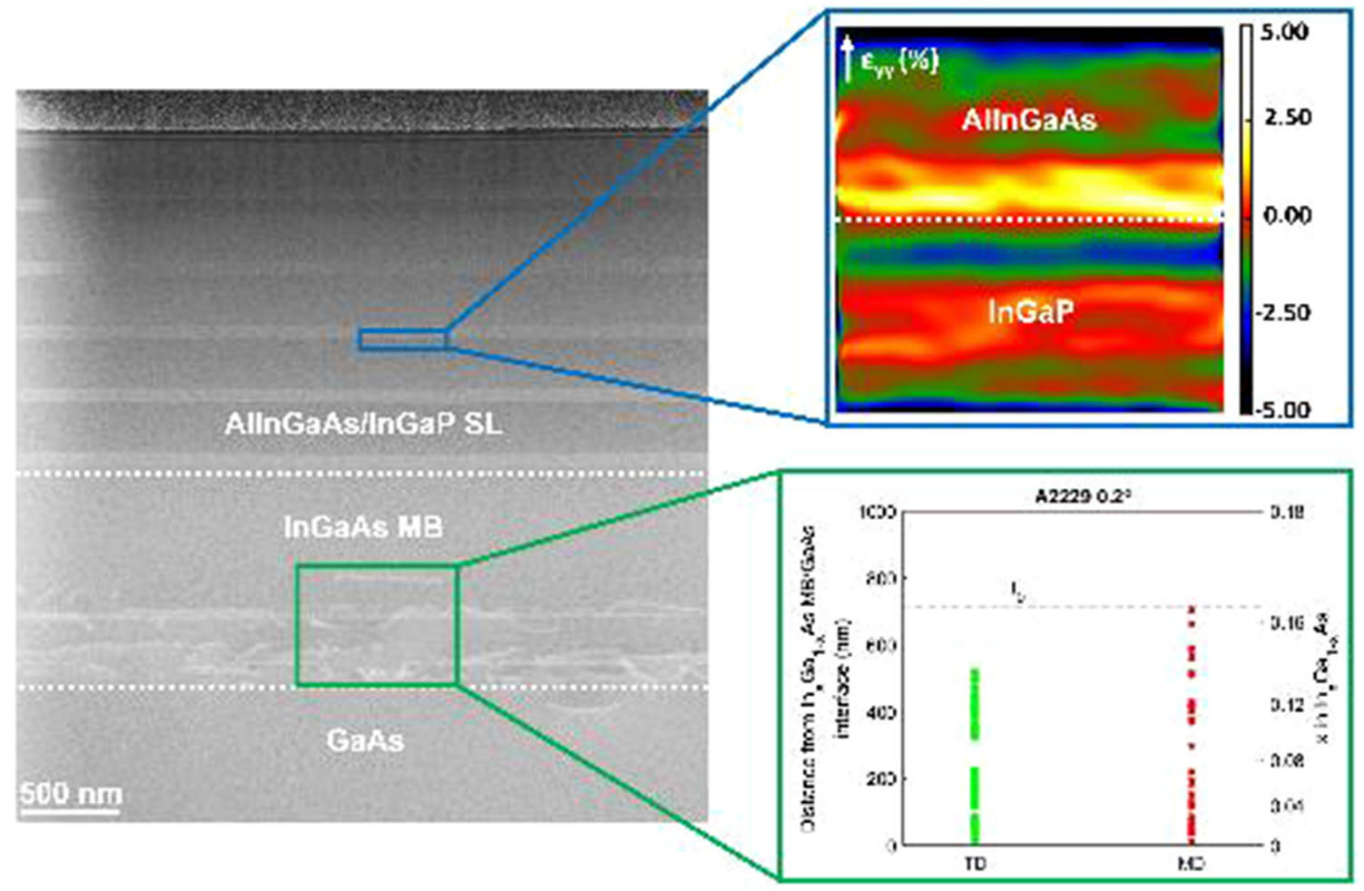

**Supplementary Information:**

The online version contains supplementary material available at 10.1007/s10853-023-08597-y.

## Introduction

Metamorphic buffers (MB) have been extensively used in the last couple of decades to design novel photonic and optoelectronic devices. Of particular interest has been the use of metamorphic buffers in strained In_x_Ga_1-x_As, lasers on GaAs substrates for telecommunications purposes [1–6]. Compared to the current InP substrates, GaAs is less brittle [7], less sensitive to temperature changes [2] and is more readily available in large wafer sizes [8]. InGaAs quantum wells (QWs) have been successfully introduced as the active region and used to tune specific wavelengths, e.g. 1.3 µm and 1.5 µm, and are thought to have less potential performance reproducibility issues when compared to their quantum dot laser counterparts [8]. However, due to constraints around the In concentration and thickness of the QW [9], commercial InGaAs QW on GaAs lasers performance is usually limited to 1.2 µm. A potential solution to this is to reduce the difference between the lattice constant of the substrate and the subsequent layers which can be achieved using a MB layer.

MBs are layers in which the composition of the alloy is gradually changed, serving as a bridge between the substrate and the subsequent layers, and hence allowing to tailor the in-plane lattice parameter of the subsequent layers. This effectively reduces the defect density, such as misfit (MD) and threading dislocations (TD) that arise at the buffer/substrate interface and is known to greatly affect the bandgap [10, 11], optical [12] and electronic [13, 14] properties of the laser.

MBs has proven to be an effective approach to decrease the defect density and relax the strain in a controlled manner, presenting an opportunity to control how the strain is distributed to the next layers and thus, offers exciting possibilities for band structure engineering. There are different chemical grading types of MB including uniform [15–17], step graded [18–20] and continuously graded [21–23]. The continuously graded approach could be linear [20, 24] or parabolic [25, 26], and generally speaking it allows more flexibility in the growth, tailoring the strain relaxation. Importantly, the parabolic profile has been shown to be less sensitive to variations in the MB thickness [22] and it has been shown to confine the defects further away from the active region [27, 28] thus, making it a more promising approach for strain tuneability.

Parabolic-graded MBs for metamorphic InGaAs QW on GaAs lasers have been recently explored for telecommunication applications at 1.3 µm. Gocalinska et al., found that for a series of different parabolic InGaAs MBs on GaAs or ‘stack designs’, the management of strain in the MB under different stack designs, with some of the findings being rather unexpected [26]. For a full GaAs metamorphic structure, several different stack designs were tried with the optimal design giving an operating laser with low lasing threshold and emission extending up to 1.36 µm [29]. Parabolic-graded MBs have also been used for InP substrate-based devices. Ye et al*.*, fabricated a photodiode operating at 2 µm with a low bit error rate which would be suitable for “laser imaging, detection, and ranging” (LIDAR) applications [30].

Indeed, MBs represent a unique opportunity for the design of novel semiconductor devices. However, the number of reports on the defect and strain distribution as a function of the architecture is limited. Typically, reports focus on the device performance or surface roughness in the metamorphic buffer and often these do not evaluate the strain levels in subsequent layers and distribution of dislocations in the MB. Strain is known to have a significant impact on the bandgap [31], which can in turn affect device performance. Therefore, establishing a connection between the strain distribution across the layers can facilitate the optimisation of metamorphic laser performance. In this paper we present a systematic study into the MB of several architectures on GaAs (001) substrate for metamorphic lasers, and a full GaAs metamorphic laser recently reported [29], via transmission electron microscopy techniques. We map the distribution and density of dislocations in the MB, with respect to In concentration. Furthermore, we present an overview on the localised strain in subsequent layers, comparing the overall strain profile of the different layers that would be used as cladding in a full laser structure. This provides an insight into the difference strain management while changing architecture/stacking for the GaAs-based metamorphic In_*x*_Ga_1-*x*_As QW lasers.

## Experimental

### Sample growth and preparation

The sample here investigated were grown using metal organic vapour phase epitaxy (MOVPE) with full growth conditions presented elsewhere [29, 32]. All precursors had purity grades at the highest of what is commercially available, for example Arsine is used at a commercial grade called Megabit III, and metalorganics were acquired by different producer, with similar quality and commercial grade availability (e.g. Optoelectronic Grade, EpiPure, and EpiGrade). Further details and discussion can be found in previous work [33–35]. Cross-sectional TEM lamellae were prepared using a TESCAN Lyra 3 dual beam FIB/SEM by standard in situ lift out procedure [36].

Electron Microscopy techniques: Transmission electron microscopy (TEM) and scanning TEM (STEM) were conducted using a Thermofishcher Talos F200-X at 200 kV fitted with a field emission gun (FEG) and four in-column Super-X energy dispersive X-ray spectrometer (EDX) detectors having a total collection angle of ∼0.9 sr. Dislocations in the MB were analysed using weak-beam dark field (WBDF) imaging mode [37]. Determination of **g** vectors was done by comparing experimental diffraction patterns to simulated using data from Giesecke and Pfister [38] in Single Crystal™ Software [39]. The number of MDs and TDs was assigned based on previous work [40, 41]. Dislocation density in the MB was measured using the line-length method [42]. The length was extracted from the annular dark field (ADF)-STEM images, using a stamp filtering process similar to the method reported elsewhere [43] with density calculated from the derived length, assumed thickness of the lamella and total area of the STEM image using an in house MATLAB script of the line-length method. The atomic fraction (at.%) profile was calculated from the EDX data, using the Kα peaks of Al, As and Ga, with Lα peaks used for In. Background correction was done with a multipolynomial function modelled as parabolic function with a Brown-Powell Ionization cross-sectional model [44]. Quantitative strain mapping was calculated using geometric phase analysis (GPA) on the ADF-STEM images using a Gatan script from Rouvière [45] and Strain +  + [46], both of which are based on theory from Hÿtch et al*.* [47]. The g-vectors tested were **g** = **002** and **g** = $\mathbf{2\overline{2 }0}$ with a comparison of **g** = **004** and **g** = $\mathbf{2\overline{2 }0}$ also investigated. Theoretical strains were calculated with lattice parameters derived from Vegard’s law [48].

## Results and discussion

### Sample overview

**Figure 1:**
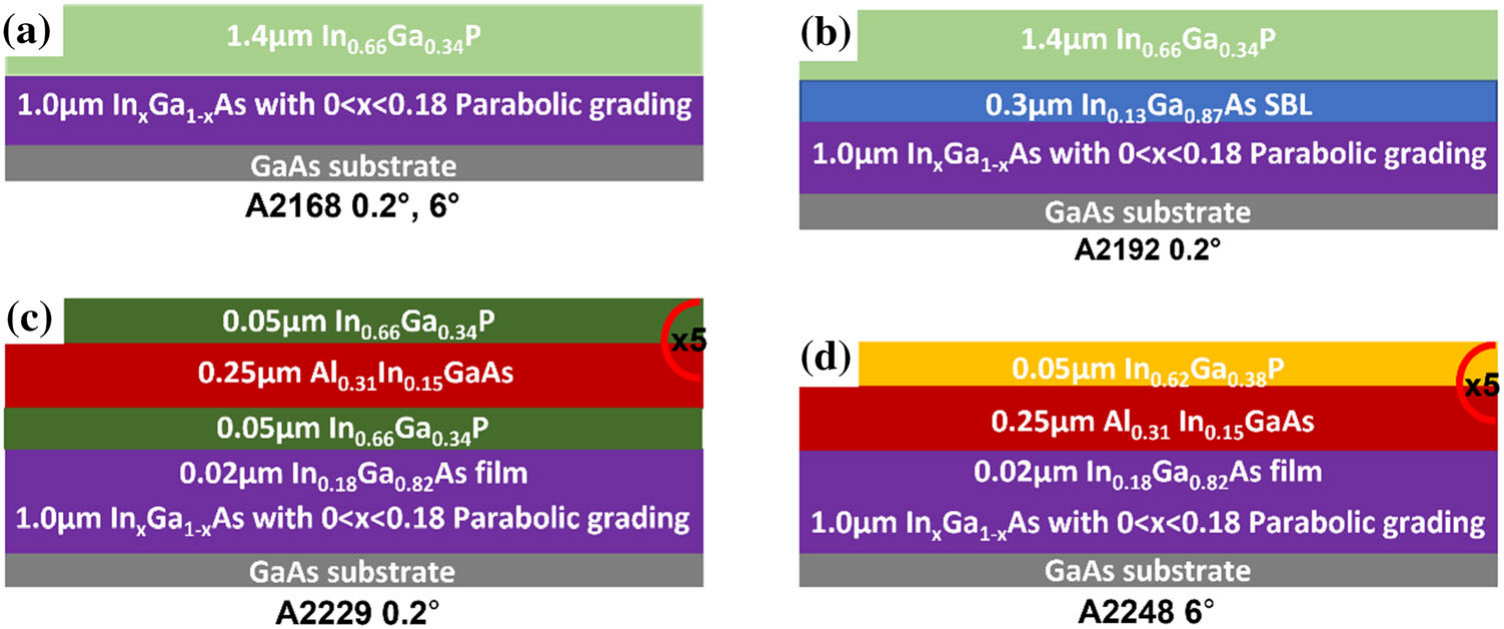
Schematic overview of five samples, with nominal thickness. **a** A2168 0.2° & 6° **b** A2192 0.2°, **c** A2229 0.2° and **d** A2248 6°. 0.2° and 6° represent misorientation towards [1 1 1]A GaAs plane. The layers are colour coded: grey represents the GaAs substrate, purple represents the In_*x*_Ga_1-*x*_As MB layer, blue is the In_0.13_Ga_0.87_As SBL, light green is the 1.4 µm In_0.66_Ga_0.34_P film, green/orange in **c** and **d** is the latticed matched/tensile strained InGaP layer in the SL samples, respectively, and dark red the Al_.31_In_0.15_GaAs layer in the SL samples. × 5 indicates the SL repetition

All samples here investigated consist of a GaAs (001) substrate misoriented towards [1 1 1]A (or also referred to as the (1 1 1) plane which terminates ideally with Ga atoms [49]) by either 0.2° or 6° on a parabolic-graded In_*x*_Ga_1-*x*_As MB with an In concentration varying from 0 < *x* < 0.18 and a nominal thickness of 1 µm. The profile of the MB is similar to the design described by Müller et al*.* [25]. After the MB, the samples have different stacking, as described schematically in Fig. 1. From bottom to top, the first sample has a GaAs substrate, the 1 µm In_*x*_Ga_1-*x*_As MB and 1.4 µm In_0.66_Ga_0.34_P film (Fig. 1a). This sample is referred to as A2168 and two variations in this are investigated, 0.2° and 6° misorientation of the GaAs substrate towards [1 1 1]A.

The second sample contains a strain balancing layer (SBL) of 0.3 µm In_0.13_Ga_0.87_As placed between the In_*x*_Ga_1-*x*_As MB and the 1.4 µm In_0.66_Ga_0.34_P film. The sample also has a 0.2° GaAs substrate misorientation, here referred as A2192 0.2°. SBLs have been reported to aid reducing surface roughness, improving film quality [26].

An alternative approach to control the strain and reduce dislocation formation in the active region is the growth of superlattices (SL) [50, 51]. Two SL structures investigated in this study consist of a GaAs substrate with the In_*x*_Ga_1-*x*_As MB (identical to all previous samples), along with a 0.02 µm thick layer of In_0.18_Ga_0.82_As on top of the MB. The two alloys used for the SLs here investigated are InGaP and Al_0.31_In_0.15_GaAs repeated five times (Fig. 1c and d). The first SL structure is lattice matched (LM) with a 0.2° misorientation towards [1 1 1]A and a 0.05 µm In_0.66_Ga_0.34_P layer on top of the In_0.18_Ga_0.82_As layer, followed by a 0.25 µm Al_0.31_In_0.15_GaAs layer which is the basis the for the SL, with 5 repetitions of the In_0.66_Ga_0.34_P and Al_0.31_In_0.15_GaAs. The top of the structure has a 0.05 µm In_0.66_Ga_0.34_P layer. This sample is referred as A2229 0.2°.

The second SL sample is intentionally strained. The sample denoted as A2248 6° follows the same MB structure as sample A2229 0.2° (Fig. 1c) with the difference that after the MB, the SL consists of a 0.25 µm Al_0.31_In_0.15_GaAs and 0.05 µm In_0.62_Ga_0.38_P repeated 5 times (Fig. 1d). In this design, the In_0.62_Ga_0.38_P is under tensile strain (TS) with respect to the Al_0.31_In_0.15_GaAs. For comparison, we also refer to a full GaAs-based parabolic-graded In_x_Ga_1-x_As MB laser structure previously investigated by Mura et al*.* [29], referred here as A2398 6° and further described in SI (see Fig. S1). Apart from being a full laser structure, the lower cladding consists of a Al_0.31_In_0.15_GaAs/TS In_0.62_Ga_0.38_P SL, similar to A2248 6° (Fig. 1d). It should be noted that the measured thickness of layers such as the MB, can deviate from the nominal thickness (see Table S1).

**Figure 2:**
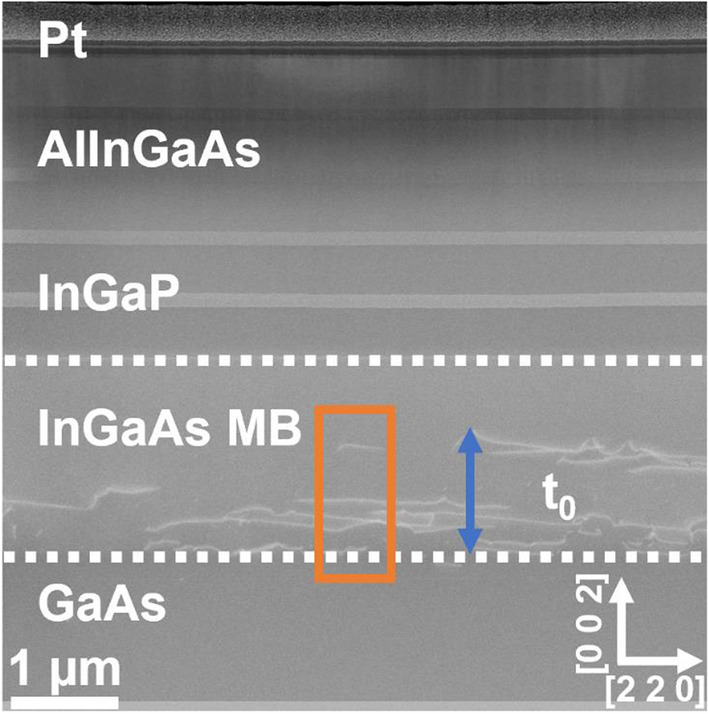
ADF-STEM overview of sample A2248 6° viewed down [1 1 0] zone axis. The blue arrow refers to the distance here referred as *t*_0_ (distance between In_*x*_Ga_1-*x*_As MB/GaAs interface and last observed MD), and the orange box represents the area from which the EDX profile shown in Fig. 2 was acquired. The white dotted line represents the interface between layers before and after the MB

Figure 2 displays a representative cross-sectional ADF-STEM image of the Al_0.31_In_0.15_GaAs/TS In_0.66_Ga_0.34_P SL sample (A2248 6°). ADF-STEM images for all samples here investigated are presented in the SI (see Fig. S2). The most striking characteristic is the presence of dislocations within the MB layer, apparent by their bright contrast. Given that one main purpose of the parabolic MB layer is to control strain relaxation and contain dislocations, it is then reasonable and relevant to investigate the relationship between the In concentration across the MB and the position, and density, of dislocations.

### Dislocations analysis

The MB here studied is In_x_Ga_1-x_As, in which as mentioned before, the concentration of In will vary as function of thickness, in a parabolic manner. As the In concentration changes so does the lattice parameters and thus, the strain is intimately linked to In concentration. This means that the dislocation density and their spatial distribution within the MB can be considered as a direct result of the changes in the In concentration. Therefore, it is of interest to investigate the relationship between In concentration and dislocations distribution in the MB.

The atomic concentration was measured by EDX as described in the experimental section. The area around the dislocation area, at the bottom of the MB, analysed for all samples is highlighted in Fig. 2. As expected, the higher dislocation density is located near the In_x_Ga_1-x_As MB/GaAs interface. However, an interesting aspect is to evaluate how far into the thickness of the MB (towards the top of the structure) dislocations can be found. In this work, we measured the distance at which the last dislocation is observed, for each sample, from the In_x_Ga_1-x_As MB/GaAs interface and refer to this distance as *t*_0._ This was measured using a combination of ADF-STEM and EDX (detailed in SI). Next, the measured In concentration is compared to the nominal In concentration to see if the In at.% is reasonable for the *t*_0_ measured. It is documented in literature that for the parabolic In_*x*_Ga_1-*x*_As-graded MB used [25, 26], the general expression for the nominal mole fraction or concentration of In at any thickness (*x*(*t*)) can be expressed in terms of the total thickness of the MB (*T*), initial concentration (*x*_in_) and the desired final In concentration (*x*_*f*_):

1 $$x\left(t\right)= {(x}_{f}-{x}_{\mathrm{in}})\left[1-{\left(1-\frac{t}{T}\right)}^{2}\right]+{x}_{\mathrm{in}}$$

The MB is In_*x*_Ga_1-*x*_As with a concentration of 0 < x < 0.18, and total nominal thickness of 1 µm. Thus, *x*_in_ takes the value of 0 and *x*_*f*_ is assumed to be ~ 0.18 and *T* = 1000 nm.

**Figure 3:**
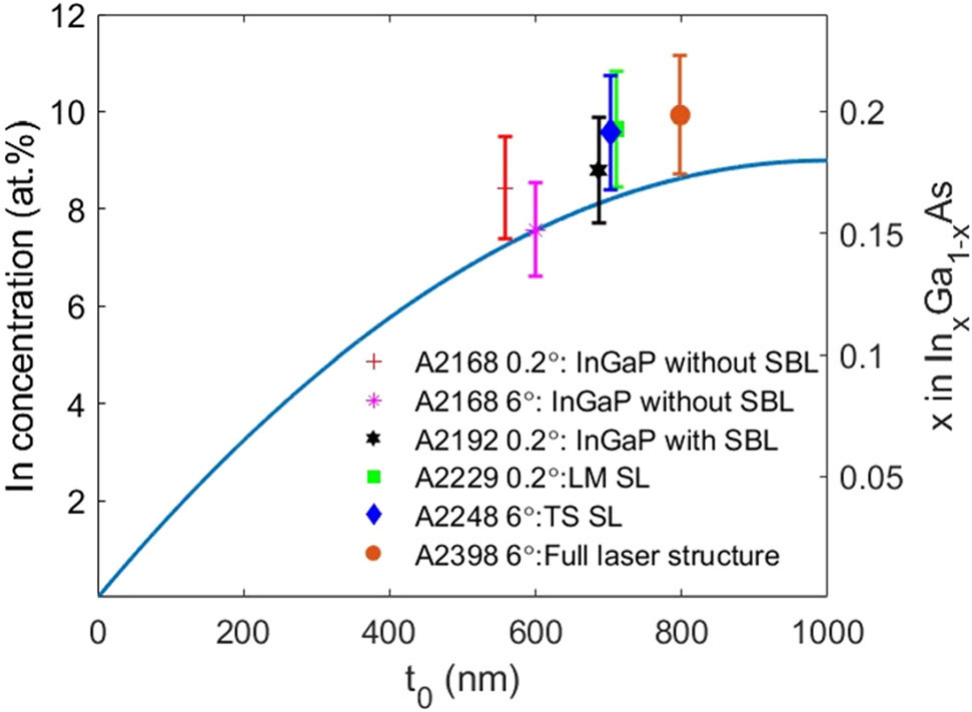
In concentration as a function of distance from the In_*x*_Ga_1-*x*_As MB/GaAs interface *t*_0_. The blue solid represents the theoretical In concentration values using Eq. 1. Six points are measured for the In at.% (left *y* axis), the corresponding *x* in In_*x*_Ga_1-*x*_As (right y axis) and t_0_, for samples A2168 0.2° (red point), A2168 6° (magenta point), A2192 0.2° (black point), A2229 0.2° (green point), A2248 6° (blue point) and A2398 6° (orange point). Tabulated values of t_0_ and In at.% provided in Table S2

Figure 3 plots *t*_0_ (the distance furthest from the In_x_Ga_1-x_As MB/GaAs interface at which dislocations can be observed) as a function of the In concentration for all samples, as well as the nominal/theoretical concentration. We can observe that the samples fit broadly within the theoretical In concentration value from Eq. 1. From this plot we can directly compare the different samples and make some interesting observations:

- The samples with the lowest t_0_ are the 0.2° and 6° misoriented samples consisting of the In_*x*_Ga_1-*x*_As MB and 1.4 µm In_0.66_Ga_0.34_P film (A2168). From these, the dislocations are found slightly higher up the MB in the sample with the larger misorientation A2168 6° (599 ± 10 nm) vs A2168 0.2° (558 ± 10 nm). A larger *t*_0_ means that the dislocation appear over a wider area, hence providing greater strain relaxation and in turn less strain in layers following the MB [25]. Considering the thickness of In_*x*_Ga_1-*x*_As MB (1.11 ± 0.01 µm for A2168 0.2° and 1.14 ± 0.01 µm A2168 6°), the larger thickness in A2168 6° could also account for the higher *t*_0_ of this sample compared to that of A2168 0.2°. This can be significant as it has been stated that increasing the MB thickness could increase *t*_0_ and vice versa [25].
- The next sample is that with the SBL layer (sample A2192 0.2°) for which the t_0_ (686 ± 10 nm) is higher compared those without the SBL (558 ± 10 nm for A2168 0.2° and 599 ± 10 nm for A2168 6°). In this case, the measured MB thickness for A2192 0.2° is 1.08 ± 0.01 µm, 0.3 µm thinner than A2168 0.2° (1.11 ± 0.01 µm) and 0.6 µm thinner than A2168 6° (1.14 ± 0.01 µm).
- The SL samples exhibit a greater *t*_0_ compared to all of the other samples. The LM sample (A2229 0.2°) has a t_0_ = 711 ± 10 nm, while the TS samples (A2248 6° and A2398 6°) have *t*_0_ values of 703 ± 10 nm and 798 ± 10 nm, respectively. Comparing the measured MB thickness of A2229 0.2° (1.16 ± 0.01 µm) and A2248 6° (1.11 ± 0.01 µm), we can see that despite the noticeable differences in thickness, the values of *t*_0_ are very close to each other, which would mean that greater MB thickness leading to higher *t*_0_ is not applicable here. However, it is clear that the full metamorphic laser structure (A2398 6°) has a significantly larger MB thickness (1.22 ± 0.01 µm) not only with to the other SL samples but also the In_0.66_Ga_0.34_P samples (A2168 and A2192 0.2°). Thus, possible explaining the reasons for the highest *t*_0_ observed in A2398 6°.

The large variation in *t*_0_ amongst the samples is perhaps surprising, given that the MB for all samples can be considered nominally similar. The measured thickness of the MB layer for all samples differ slightly from the nominal 1 µm (see Table S1) and this could be related to small differences in the associated MOVPE growth [52]. However, it is not clear to the authors why t_0_ varies by ~ 200 nm. The MB thickness for A2192 0.2° (1.08 ± 0.01 µm) is ~ 30 nm lower than both A2168 samples; however, the t_0_ is much higher. Similarly, the increasing t_0_ cannot explain the observations of A2192 0.2° as the MB thickness for A2192 0.2° is lower in comparison with both A2168 samples. In summary, the *t*_0_ values are higher in the SL and the measured In concentration is very close to the expected In concentration at the measured *t*_0_ value.

Having measured the maximum distance at which dislocations can be found in the MB layer (*t*_0_), we now consider the dislocation density. Table 1 shows the measured dislocation density observed in the In_x_Ga_1-x_As MB up to *t*_0_. Four different areas were looked at in the MB region with lowest and highest dislocation density referring to lowest/highest dislocation density recorded. It should be noted that in all samples the MDs seem to be mainly contained within two regions, evoking reports showing that MDs occur in two waves [53, 54]. The first wave is considered to occur at the point where MDs become energetically favourable and provide minimal strain relief, known as critical thickness [55]. The second wave could be formed significantly further away from this critical thickness after which the layer relaxes completely [53].

**Table 1:** Dislocation density in MB region, between In_*x*_Ga_1-*x*_As MB/GaAs substrate interface and *t*_0_ for A2168 0.2°, A2168 6°, A2192 0.2°, A2229 0.2°, A2248 6° and A2398 6°

Sample	Lowest dislocation density (cm^−2^)	Highest dislocation density (cm^−2^)
A2168 0.2°	3.20 × 10^8^	5.72 × 10^9^
A2168 6°	6.68 × 10^8^	3.18 × 10^9^
A2192 0.2°	5.11 × 10^9^	5.96 × 10^10^
A2229 0.2°	7.96 × 10^8^	1.07 × 10^10^
A2248 6°	1.60 × 10^9^	2.71 × 10^10^
A2398 6°	6.24 × 10^8^	4.87 × 10^9^

From this it can be seen that all samples have a dislocation density between ~ 10^8^ and 10^10^ cm^−2^ in agreement with previous reports for similar systems [26, 56]. Comparing the samples with the simpler design, those with the In_0.66_Ga_0.34_P film after the MB (samples A2168 and A2192 0.2°), an immediate observation is that the sample containing the SBL layer (A2192 0.2°) exhibits a dislocation density one order of magnitude higher when compared to the samples without the SBL. A reason for the difference in density could be explained by the inclusion of the SBL itself. Previous work has shown for two parabolic-graded MB (one MB below and one MB above the SBL) that the dislocation density decreases with the inclusion of the SBL [26]. Conversely, the dislocation density with a single parabolic-graded MB with an SBL was shown to have similar density compared without the SBL [26]. An alternative consideration is that the thickness of the SBL (in the case of being too thick) can have a detrimental effect. For this buffer a thickness above 0.30 µm was considered to lead to roughening [8].

Similarly, the dislocation density is higher for the SL samples (A2248 6° and A2229 0.2°) compared to the A2168 samples. To the authors knowledge while no specific densities have been quoted for this SL system in a parabolic-graded InGaAs MB, it has been established that both the TS and LM SL in InGaAs/GaAs can reduce dislocation density by one order of magnitude [51] and there is no consensus in the literature that one type of SL would lead to significantly lower density than the other. In other words, it is not surprising that both samples A2229 0.2° and A2248 6° have similar dislocation densities. This however do not explain why the SL samples have a higher density compared to the A2168 samples. Regarding the full metamorphic laser structure (A2398 6°) we observe that the dislocation density is one order of magnitude lower than both A2229 0.2° and A2248 6° despite using a similar TS SL design based off A2248 6°. These could be explained by the fact that the SL in A2398 6° was further optimised by using linear ramping (gradually increasing the Al concentration in each AlInGaAs layer in the SL) and reducing the number of units in the SL [29].

For dislocation measurements, it is important to consider the lamella thickness and the method used. Looking at Figs. 1 and S1, we can clearly see that dislocations are more visible in the SL samples and the In_0.66_Ga_0.34_P film with the SBL (A2192 0.2°) but not as clear in the In_0.66_Ga_0.34_P film samples without the SBL (A2168). This could mean an underestimation of the true density in the A2168 samples, as a thicker lamella might exhibit more dislocations than a thinner lamella. A second point to consider is the method which uses stamp filtering on identifying dislocations using contrast (*i.e.* white lines observed in Figs. 1 and S2) across the image. The risk here is that parts of the image that are not dislocations may be included and conversely, dislocations may not be identified and in turn be omitted. The filtering procedure included manual selection of dislocations regions to help exclude areas that were not dislocations.

It is known that dislocations appear as a strain relief mechanism, and their distribution provides a starting point to further understand strain relaxation mechanisms in strained layers. It has been theoretically shown that in compositionally graded layers pinning can be greatly reduced, and there is a much larger residual strain at the surface with reduced strain deep inside the graded layer [27]. The classification and multiplication of dislocations has been extensively studied for GaAs-based systems. Mainly, there are MDs which lie parallel to the interface and can be classified as 60° and 90°. 60° MDs glide on {111} type of planes [57, 58], and lie on the $\left[1 1 0\right]$ or $\left[1 0 \overline{1 }\right]$ (for *α* and type *β* dislocations, respectively, with distinct mobility which results in asymmetric distribution of dislocations [57]). Their Burgers vectors are of the type $\frac{a}{2}\left[1 0 1\right]$, $\frac{a}{2}\left[0 1 \overline{1 }\right]$, $\frac{a}{2}\left[1 0 \overline{1 }\right]$, $\frac{a}{2}\left[0 1 1\right]$ where $a$ is the lattice constant of InGaAs [58–60]. Edge dislocations, or 90° MDs, lie on the (001) plane and can have Burgers vector of either $\frac{a}{2}[1 \overline{1 } 0]$ or $\frac{a}{2}[1 1 0$] and can be formed by two 60° MDs interacting with one another [40]. Additionally TDs can form as a result of MDs which do not terminate at the surface of a crystal [41]. TDs can act as non-radiative recombination centres [56] where the recombination of the electron and hole during the electron transition leads to formation of a phonon. This results in unwanted generation of heat energy, an undesired effect in a laser. It should be noted that previous reports have also suggested that edge dislocations can act as non-radiative recombination centres [61]. The classification as such of the dislocations is not the scope of this work and we investigate the distribution of MDs and TDs within the MB layer, to gain a better understand how the strain is relaxed towards the next interface.

**Figure 4:**
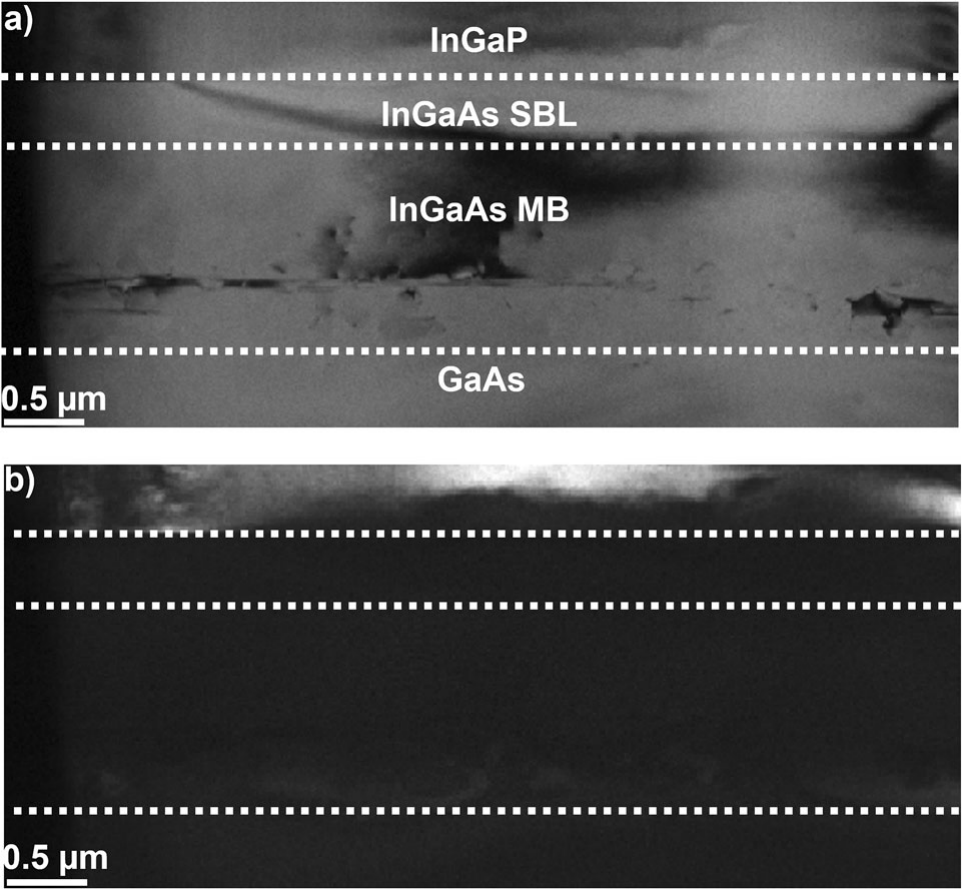
WBDF analysis for sample A2192 0.2°. Bright field TEM image of A2192 0.2° **a** and corresponding WBDF field image with g_220_ **b**. Images viewed down [1 1 0] zone axis

WBDF analysis was performed, using the invisibility criterion **g**.(**b** × **u**), where **g** is the diffraction vector, **b** is the Burgers vector of the dislocation and **u** is the line direction of the dislocation [62]. Figure 4 shows a representative WBDF analysis for sample A2192 0.2° where most dislocations lie parallel to the [2 2 0] direction, in agreement to previous reports [63, 64]. Considering that the same nominal recipe for MB is used for all samples, similar results can be expected from the samples studied here (see Figs. 1 and S2).

**Figure 5:**
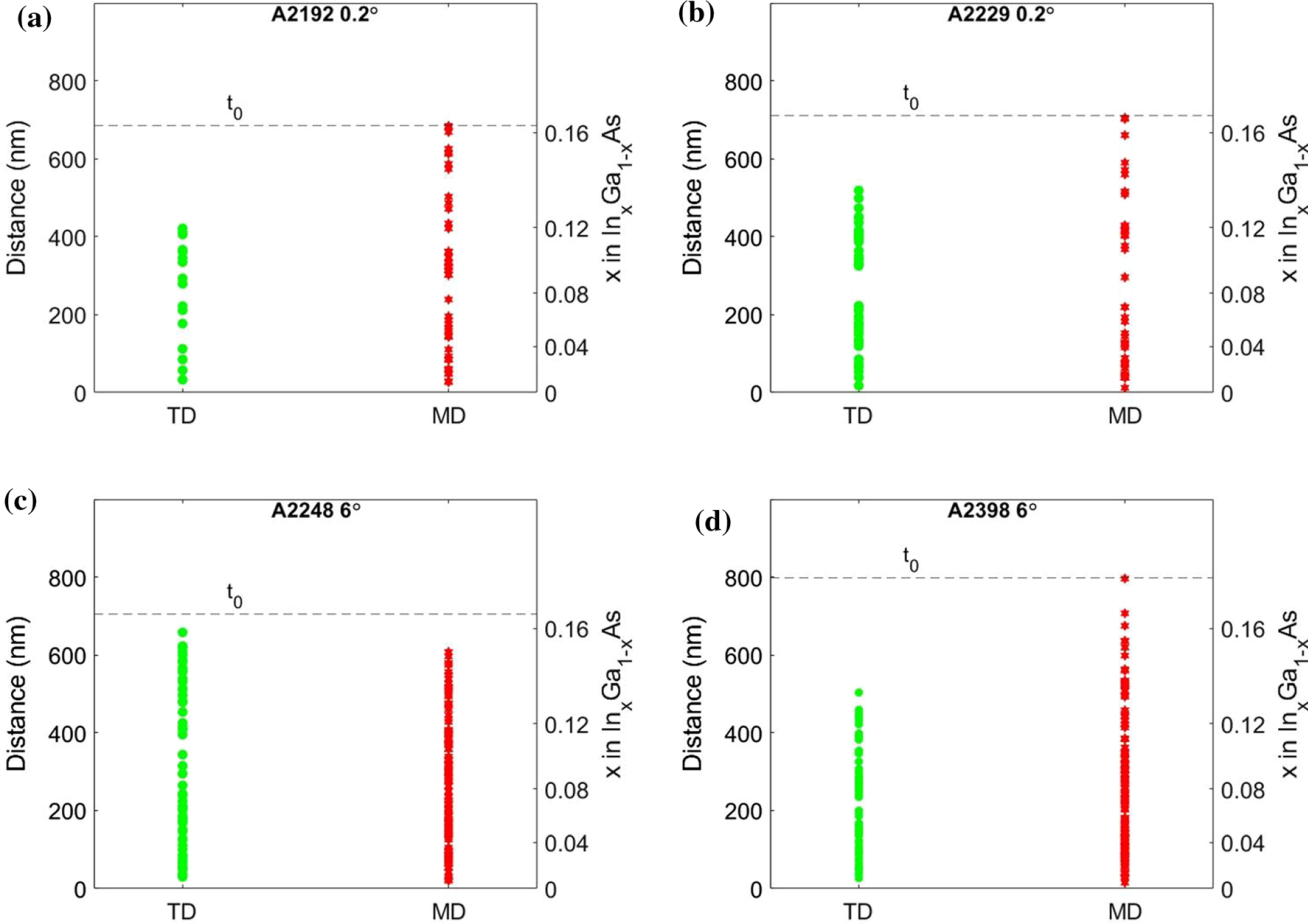
Distribution of MDs (red points) and TDs (green points) in the MB for all samples up to *t*_0._ for A2192 0.2° **a**, A2229 0.2° **b**, A2248 6° **c** and A2398 6° **d**. The distance is measured from the InGaAs MB/GaAs substrate interface. The dotted line represented *t*_0_. Full details on the data points used for each plot can be found in Table S3

Using the **g**_**220**_ vector, all of the dislocations become invisible, both 60° and 90° MDs, which is in agreement with previous results [58, 65]. WBDF using **g**_**002**_ (see SI) suggests asymmetry within the dislocations which could be a consequence of the mistilt of the GaAs (001) towards the [1 1 1] A plane that leads to a change in the shear strain on the glide plane of the MDs, favouring certain MD formation [66] or can be indicative of the asymmetry previously discussed in 60° MDs. Thus, we can confirm that within the limitations of a cross-sectional analysis, most of the dislocations here observed are MDs with a few TDs. It should be noted that it is possible that both TD/MD components could be present such as a TD with MD [67] or two TDs that originate from a half loop of a MD [41]. Figure 5 summarises the distribution of all dislocations in the MB up to t_0_. The number of data points used for each sample and the total number assigned as MD and TD is outlined in SI.

Except for the TS SL sample (A2248 6°), it can be observed that the MDs tend to be closer to t_0_ compared to TDs and TDs appear to stop between 200 nm and 300 nm away from t_0_. This suggests that the MDs are pinning the TDs in the lower region of the MB where a lower strain level can be expected. Considering the In concentration shown in Fig. 3, we can identify approximately the In mole fraction (*x*) at which TD stop in the MB. From the plots, it suggests that: (i) for the 1.4 µm In_0.66_Ga_0.34_P sample with the SBL (A2192 0.2°) is *x* ~ 0.12, (ii) for the LM SL sample and full metamorphic laser structure (A2398 6°), *x* ~ 0.14 and (iii) for A2248 6°, *x* ~ 0.16. Comparing the points for the MDs, we have a strong indication that two waves of MDs are present, one starting near the GaAs/InGaAs MB interface and the other starting higher up, as documented in the literature [40, 53].

So far, the variation in the t_0_ is perhaps a surprising finding that could be explained by the possible variations in the nominal recipe used to grow the MB layers. However, a common aspect is that the MDs appear in two waves, with close proximity of each other and that these waves are very close to the measured t_0_. It is now important to evaluate how the strain is distributed to the layers above the MB, as the strain greatly influences the laser properties. Here, the main emphasis will be given to the growth direction [0 0 2], as this is the strain that propagates up the structure from the MB towards a lower cladding layer which comes before the active region in a metamorphic laser or any other semiconductor device.

### Strain mapping

The strain profile in the AlInGaAs and InGaP layers, immediately after the In_*x*_Ga_1-*x*_As MB, was measured by applying GPA on high-resolution ADF-STEM micrographs. It should be noted that a positive value represents compressive strain and negative values indicate tensile strain [68]. For consistency, all micrographs were analysed with the same magnification and procedure as outlined in SI. As indicated in the experimental section, the STEM micrographs used for the strain analysis are non-corrected and thus, the focus will be on the strain trend and variations between the different sample designs. The full details on the analysis and considerations for this are detailed in the SI.

**Figure 6:**
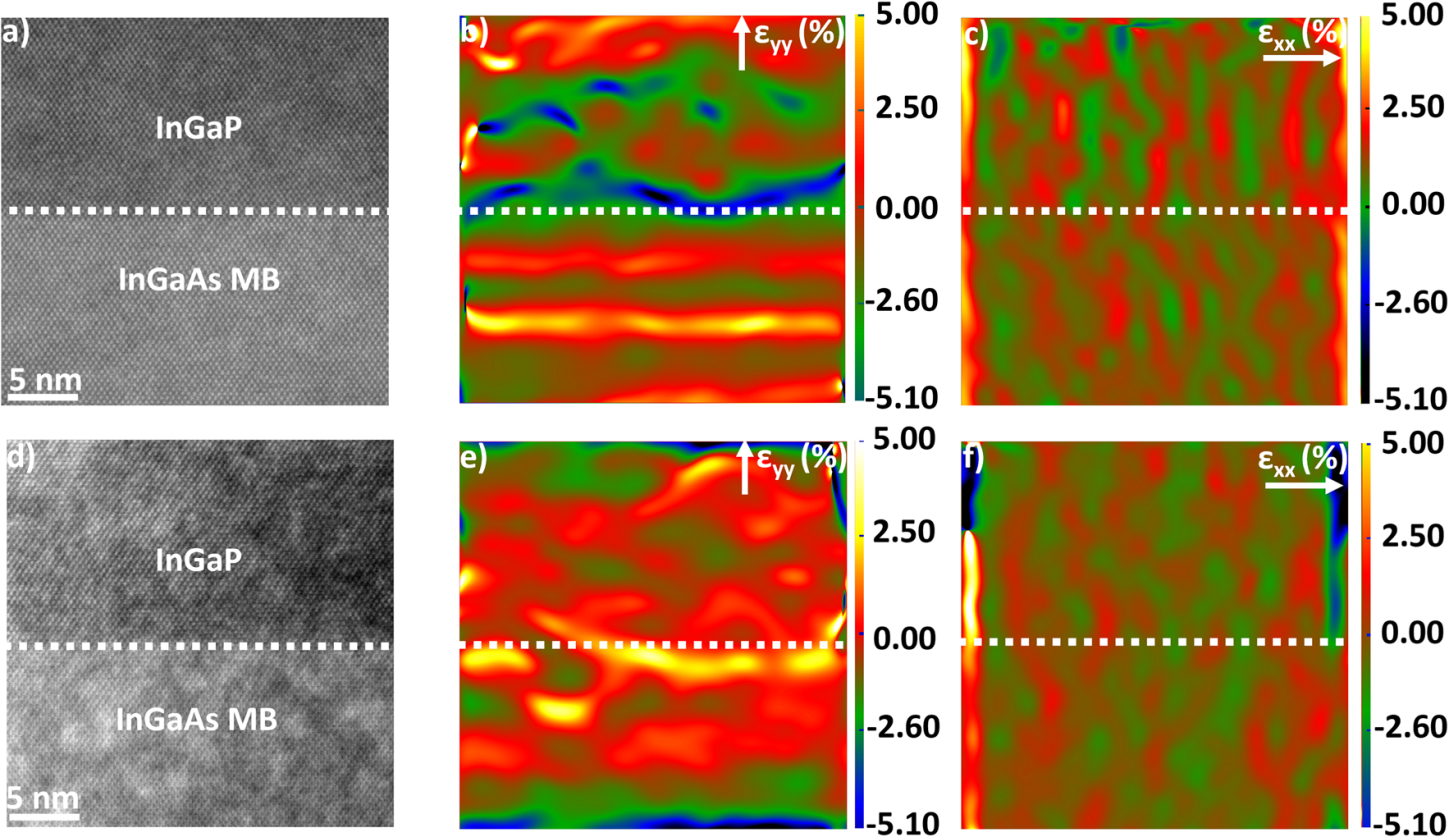
High-resolution STEM of In_0.66_Ga_0.34_P/In_*x*_Ga_1-*x*_As MB interface in A2168 0.2° used for GPA **a** with ε_*yy*_ strain **b** and *ε*_*xx*_ strain **c**. HRSTEM of In_0.66_Ga_0.34_P/In_*x*_Ga_1-*x*_As MB interface in A2168 6° used for GPA **d** with *ε*_*yy*_ strain **e** and *ε*_*xx*_ strain **f**. All images viewed down [1 1 0] zone axis

**Figure 7:**
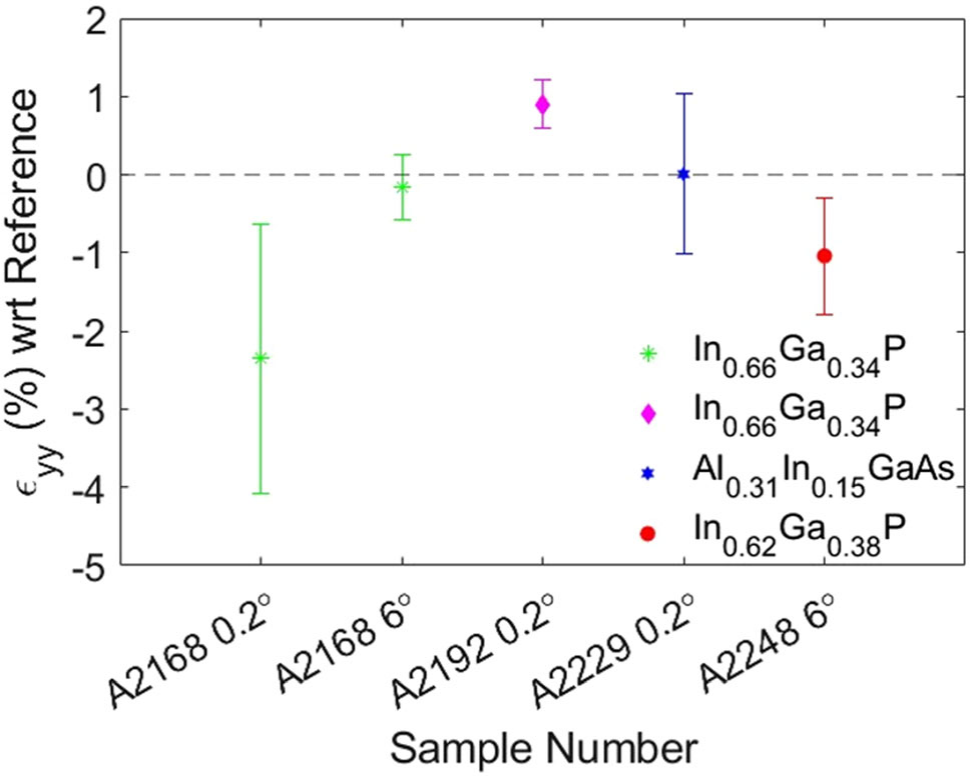
*ε*_*yy*_ strain in layers with respect to (wrt) reference regions. Strain in In_0.66_Ga_0.34_P layer in A2168 0.2° and A2168 6° measured with respect to In_*x*_Ga_1-*x*_As MB (green point), In_0.66_Ga_0.34_P layer in A2192 0.2° with respect to In_0.13_Ga_0.87_As SBL (magenta point), Al_0.31_In_0.15_GaAs layer in A2229 0.2° with respect to In_0.66_Ga_0.34_P (blue point) and In_0.62_Ga_0.38_P layer in A2248 6° (red point) with respect to Al_0.31_In_0.15_GaAs. Tabulated values of *ε*_*yy*_ can be found in Table S5

The full strain maps for all samples are detailed in SI and Fig. 7 summarises the strain along the growth direction [0 0 2] (*ε*_*yy*_). Figure 6 displays representative relative strain maps for the samples with the simpler 1.4 µm In_0.66_Ga_0.34_P film after the MB design (A2168 0.2° and A2168 0.6°) as differences in misorientation are one of the main factors influencing the strain [69, 70]. In A2168 0.2°, the strain is highest in *ε*_*yy*_ and more relaxed in the in-plane direction [220] (*ε*_*xx*_). In comparison, A2168 6° has a lower magnitude of strain in *ε*_*yy*_ (− 0.16 ± 0.42%) and higher strain in *ε*_*xx*_ (− 0.25 ± 0.04%) compared to the sample with lower 0.2° misorientation towards [1 1 1]A. Based on Vegard’s Law (see SI) and the proposed chemical composition of the sample, the theoretical strain between an In_0.66_Ga_0.34_P layer and the In_0.18_Ga_0.82_As MB would be − 0.07%. Comparing the theoretical strain values to the experimental strain, the In_0.66_Ga_0.34_P *ε*_*yy*_ component is in agreement with the predicted theoretical strain for A2168 6°. Furthermore, the tensile strain in A2168 0.2° (− 2.35 ± 1.72%) is outside the expected − 0.02% strain. The addition of the SBL layer (A2192 0.2°) has changed the strain from tensile (− 2.35 ± 1.72%) to under compressive (0.43 ± 0.29%). This is not unexpected given that the theoretical strain between In_0.66_Ga_0.34_P film layer grow on In_0.13_Ga_0.87_As is 0.36% (see SI). A further point that should be noted is the surface at the very top of the In_0.66_Ga_0.34_P layer in A2192 0.2° displays peaks and valleys (see Fig. S2C.) which were also observed in the bulk of the sample during FIB milling. This could be indicative of build-up of strain in the system and the strain relaxation not happening as effectively in sample A2192 0.2° [26]. From Fig. 3, it can be seen that A2192 0.2° has the lowest t_0_, which as discussed by Tersoff [27] would mean that more strain is present in the system at the end of the metamorphic buffer. Thus, a build-up of strain after the metamorphic buffer would be expected as reported by Romanato et al*.* [71].

As expected, the *ε*_*yy*_ value for the LM SL sample (A2229 0.2°) is minimum (0.01 ± 1.01%). This is reasonable given that Al_0.31_In_0.15_GaAs and In_0.66_Ga_0.34_P in the SL are lattice matched to each other. With regards to the sample with the TS SL (A2248 6°) the In_0.62_Ga_0.38_P layer is under tensile strain (− 1.04 ± 0.75%) in *ε*_*yy*_ relative to the Al_0.31_In_0.15_GaAs, Fig. 7. This is expected as the lattice constant of Al_0.31_In_0.15_GaAs (0.5727 nm) will be the same as In_0.66_Ga_0.34_P (as Al_0.31_In_0.15_GaAs /In_0.66_Ga_0.34_P are LM) and in turn the lattice constant of In_0.62_Ga_0.38_P (0.5710 nm) is smaller relative to that of Al_0.31_In_0.15_GaAs. This leads to tensile strain in the In_0.66_Ga_0.34_P layer (see SI). It should be stated that the measured strain (− 1.04 ± 0.75%) does not fall within predicted strain (− 0.06%). Possible reasons for discrepancy could include local differences in the chemical composition (*i.e.* not being the exact same as the theoretical concentrations used in the calculation), thickness variations in the sample [72], differences in size/location of the reference region [45, 73], and the mask size used during GPA analysis which has been demonstrated to influence accuracy [74] (as discussed in the SI).

In general, this strain analysis provides a good indication of the type of strain present in subsequent layers and clearly demonstrates the differences in the strain distribution in In_0.66_Ga_0.34_P film or AlInGaAs/InGaP SL after the MB for each architecture. That is, changing the misorientation and the inclusion of SBL affects the strain in the In_0.66_Ga_0.34_P film.

Finally, as an additional test on the strain calculations here obtained, the strain in a full metamorphic laser structure (A2398 6°) was measured to compare the GPA analysis between non-Cs and Cs corrected STEM images. In both cases the Al_0.31_In_0.15_GaAs layer was calculated to be tensile strained with respect to the InGaAs MB and both values were within the same order of magnitude (− 1.03 ± 0.03% for the non-Cs image vs − 0.92 ± 0.71% for the Cs corrected image). A further comparison between GPA scripts and reflections was carried out, and detailed in SI, supporting the conditions here used.

## Conclusions

We investigated InGaAs MB on GaAs substrate samples with different architectures and looked at the distribution of dislocation in the MB and subsequent strain in preceding layers. In general, the dislocation density was found to be one order of magnitude higher in samples with Al_0.31_In_0.15_GaAs/InGaP SL (10^9^–10^10^ cm^−2^) compared to samples with an In_0.66_Ga_0.34_P film (10^8^–10^9^ cm^−2^) after the In_*x*_Ga_1-*x*_As MB.

We have correlated the In concentration to the dislocations propagation, and identified the region above the GaAs buffer where the dislocations would relax (stop). Depending upon the sample, this was found to vary from 550 nm to 800 nm from the In_x_Ga_1-x_As MB/GaAs interface. We propose that this large variation can be partially explained by variations in the MB thickness across the samples. Importantly, it was found that there is good agreement between the theoretical predicted and the measured In concentration values at t_0_. In the In_x_Ga_1-x_As MB, it was seen that MDs are concentrated near the value of t_0_ and depending upon the architecture, it was common for TDs to end approx. 200–300 nm before the last MD. The dislocation density showed that the sample with the In_0.13_Ga_0.87_As SBL between the In_0.66_Ga_0.34_P film and In_x_Ga_1-x_As MB displayed the highest dislocation density.

The strain mapping between the MB and the subsequent layers provides a systematic insight into the local strain levels across different architectures (AlInGaAs/InGaP or in InGaP film after the MB). The common aspect is that generally the strain is largest in the growth direction whilst the system is relaxed in the out of the plane direction. And the change in misorientation towards [1 1 1]A (0.2° vs 6°) has shown to greatly reduce the strain, from tensile to no overall strain. Furthermore, the addition of an SBL changed the strain from tensile to compressive, matching theoretical predictions. As expected, there was no overall strain in the LM Al_0.31_In_0.15_GaAs/In_0.66_Ga_0.34_P SL while there was tensile strain in the In_0.62_Ga_0.38_P layer in the Al_0.31_In_0.15_GaAs/In_0.62_Ga_0.38_P SL.

Overall, these findings provide further insight into how the dislocations are distributed in the MB and the effects this has on the strain distribution across the system. Knowledge of the strain and the dislocation distribution provides a key understanding on different approaches to tailor the strain in semiconductor devices, especially metamorphic lasers.

## Supplementary Information

Below is the link to the electronic supplementary material.

Supplementary information document attached provides further details of calculations and procedures used for dislocation and strain analysis. The document also provides all figures, full table of results and additional dislocation and strain analysis that were not included as part of the main text (DOCX 14330 KB)

## Data Availability

Not applicable.
