# Supplementary material for: Dislocation and strain mapping in metamorphic parabolic-graded InGaAs buffers on GaAs
Source: J Mater Sci. 2023 Jun 7;58(23):9547–61. doi: 10.1007/s10853-023-08597-y (PMC10261241; doi:10.1007/s10853-023-08597-y)
Supplement: Supplementary file 1 — Supplementary information document attached provides further details of calculations and procedures used for dislocation and strain analysis. The document also provides all figures, full table of results and additional dislocation and strain analysis that were not included as part of the main text (DOCX 14330 KB) [file 10853_2023_8597_MOESM1_ESM.docx]

**Supplementary information**

1. Sample overview

Figure S1 indicates the full InGaAs QW/GaAs metamorphic laser structure investigated in this report which is denoted as A2398 6°. An AlInGaAs/InGaP SL design similar to A2248 6° from was used as the cladding layers for A2398 6°. The n cladding layer is doped with Zn with the Separate Confinement Heterostructure (SCH) consisting of AlInGaAs/InGaP acting as the barrier layer. A three layer multi InGaAs QW system with a GaAs control interface layer (CIL) and In_0.40_Ga_0.60_As barrier is then deposited as the active region. The rest of the laser is complete by adding a second SCH and the p-type cladding with a final InGaAs contact layer.


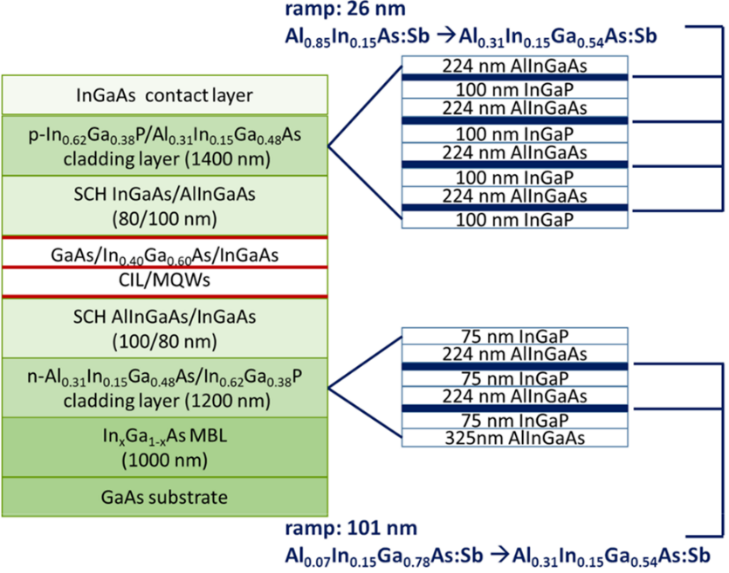


***Figure S1*** *Schematic diagram overview of A2398 6°. From “Mura EE, Gocalinska AM, O’Brien M, et. al. (2021) Importance of Overcoming MOVPE Surface Evolution Instabilities for >1.3 μm Metamorphic Lasers on GaAs. Cryst Growth Des 21:2068–2075. https://doi.org/10.1021/acs.cgd.0c01498”.*

*
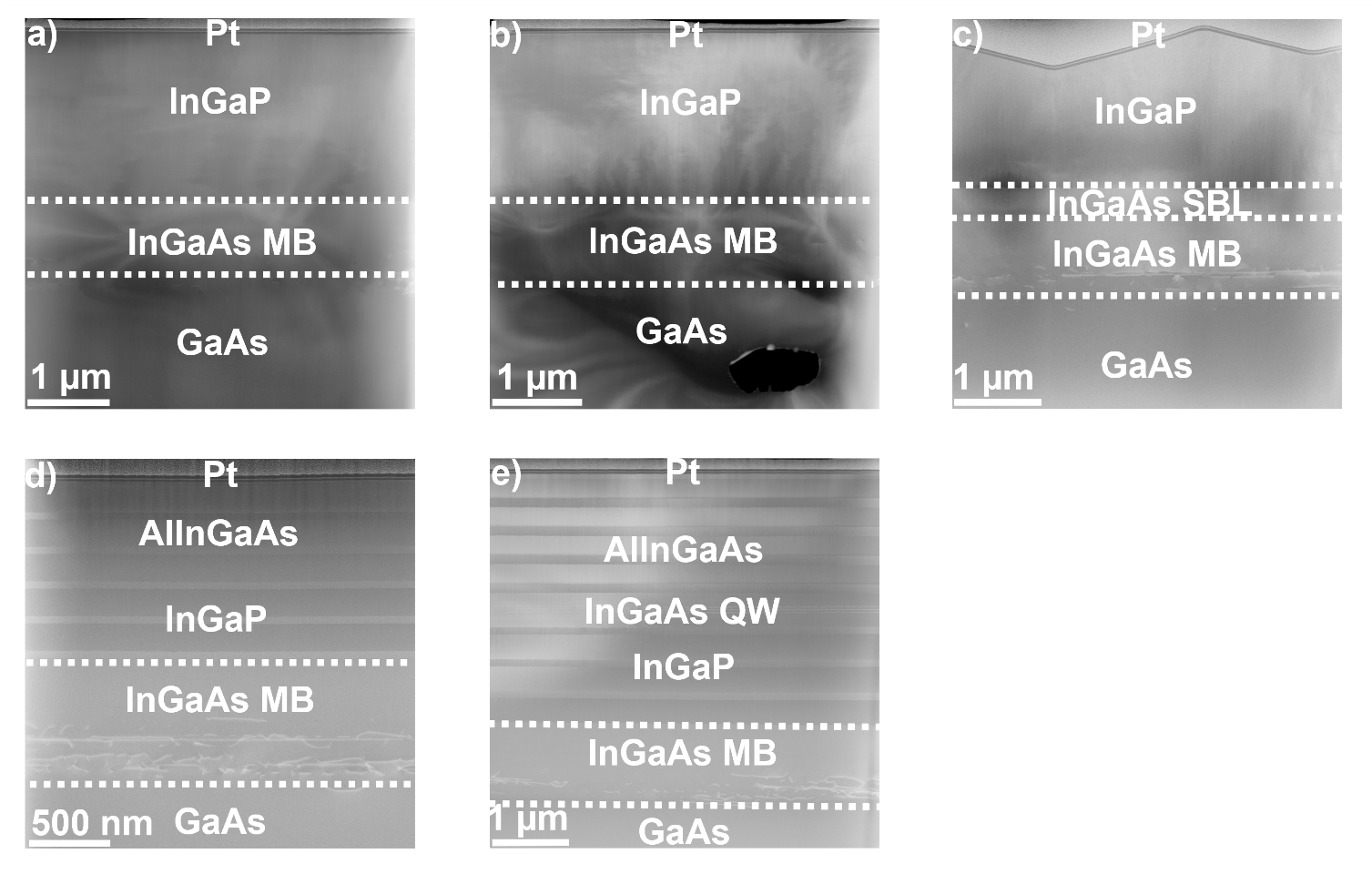
*

***Figure S2*** *ADF-STEM overview of samples A2168 0.2° (a), A2168 6° (b), A2192 0.2° (c), A2229 0.2° (d) and A2398 6° (e). All images viewed down [1 1 0] zone axis.*

Table S1 displays the In_x_Ga_1-x_As MB measured thickness for each sample here. A clear variation in the measured thickness (vs the nominal thickness, 1µm) can be observed, due to the MOVPE process reproducibility over a long period of time.

| Sample | MB Thickness  (µm) |
| --- | --- |
| A2168 0.2° | 1.11±0.01 |
| A2168 6° | 1.14±0.01 |
| A2192 0.2° | 1.09±0.01 |
| A2229 0.2° | 1.16±0.01 |
| A2248 6° | 1.11±0.01 |
| A2398 6° | 1.22±0.01 |

***Table S1*** *Measured thickness of In_x_Ga_1-x_As MB in samples A2168 0.2°, A2168 6°, A2192 0.2°, A2229 0.2°, A2248 6° and A2398 6°.*

1. Dislocations analysis

We will outline in detail how t_0_ was calculated. To derive t_0_, we use EDX to assign the location of the In_x_Ga_1-x_As MB/GaAs interface and placed on an ADF-STEM image. The EDX is required as the interface is not clearly seen directly from the STEM image. It would be expected that the In fraction would start increasing going from GaAs into the In_x_Ga_1-x_As MB (*i.e.* this would indicate In_x_Ga_1-x_As MB/GaAs the interface) as highlighted in Fig S3B.


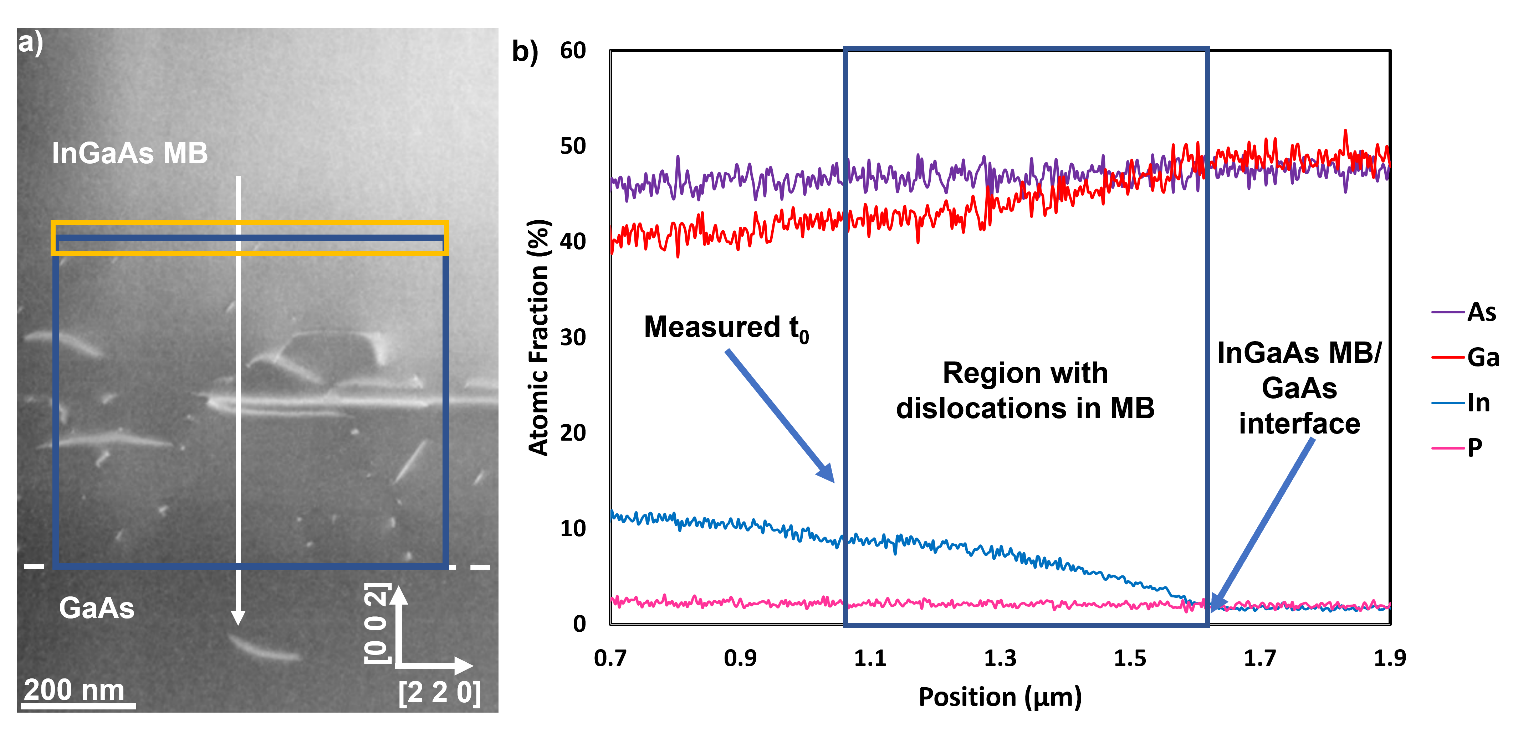


***Figure S3*** *Dislocations overview within MB. ADF-STEM of dislocations viewed down [1 1 0] zone axis in A2192 0.2° (a) and elemental atomic fraction line profile (b) from the area marked by white arrow in a). Length of blue box indicates t_0_ and gold box area where In at. % fraction at t_0_ was measured.*

The In at. % and corresponding uncertainty at t_0_ was measured drawing a box at the location of the last MD that is approx. 20nm in height and approx. 350 nm in width as illustrated in Fig S3a. The slight variation in sizes is a consequence of different magnifications used for the images.

| Sample | t_0_  (nm) | In at.% at t_0_  (%) | Measured box height  (nm) | Measured box width  (nm) |
| --- | --- | --- | --- | --- |
| A2168 0.2° | 558±10 | 8.44±1.05 | 20.58 | 354.1 |
| A2168 6° | 599±10 | 7.57±0.96 | 20.58 | 354.1 |
| A2192 0.2° | 686±10 | 8.80±1.09 | 20.38 | 358.1 |
| A2229 0.2° | 711±10 | 9.64±1.18 | 20.58 | 354.1 |
| A2248 6° | 703±10 | 9.57±1.28 | 17.47 | 349.4 |
| A2398 6° | 798±10 | 9.93±1.22 | 18.93 | 349.4 |

***Table S2*** *t_0_ and* *corresponding In atomic fraction for samples A2168 0.2°, A2168 6°, A2192 0.2°, A2229 0.2°, A2248 6° and A2398 6°.*

We will now outline in more detail how the dislocation density in the In_x_Ga_1-x_As MB was obtained. Dislocation density in the MB was derived using the line length method on STEM/TEM images. The dislocation density, ρ, in the metamorphic buffer is given by

$\rho=\frac{L_{d}}{A \times t}$ (Eq S1)

Where L_d_ is the total length of dislocations identified in the image, A is the total area of the image and t is the thickness of the sample. For this work, it was assumed that the sample had a uniform thickness of 60nm.

L_d_ was found using IMAGE J. Taking an ADF-STEM or TEM image (Fig. S4a) with a known pixel calibration we identify areas of dislocation with shapes, clearing the background to give an image as shown in Fig. S4b. We then a perform stamp filtering process, adjusting the threshold to give a binary image so that lines representing dislocations are black and those that are not (*i.e.,* background) in white in (Fig. S4c). Finally perform a skeletonise function to the binary image to yield the length of dislocations (Fig. S4d). This was repeated over 4 separate images for each sample.


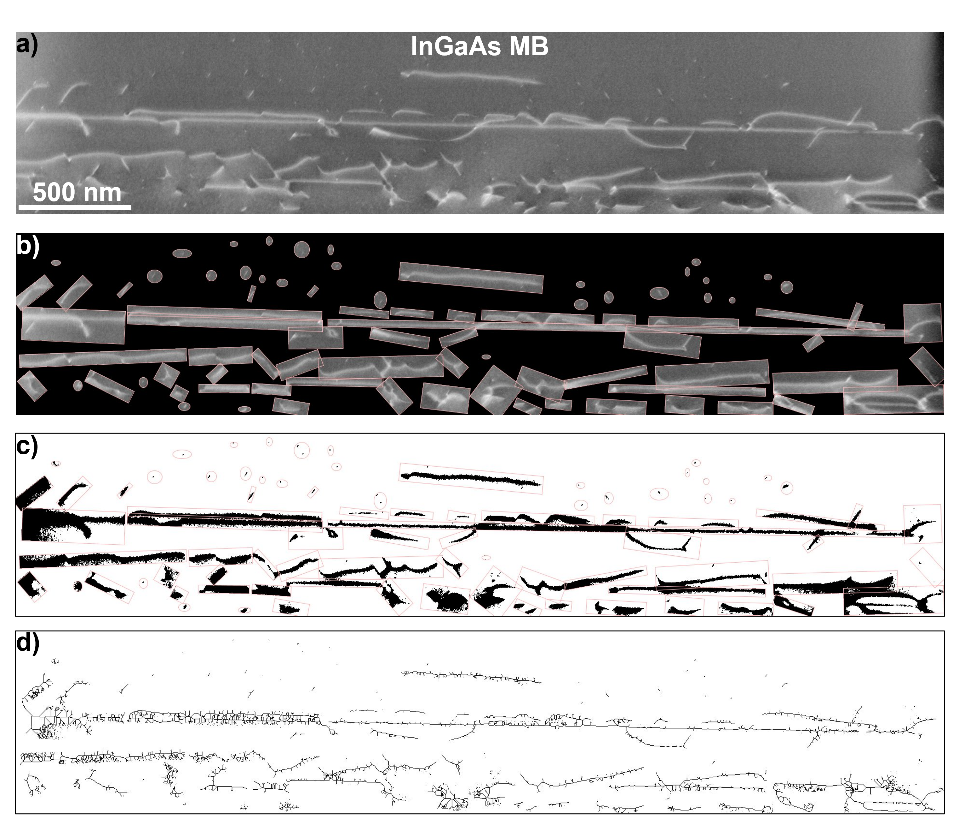


***Figure S4*** *Stamp filtering process of finding dislocations from A2248 6°. Starting with an ADF-STEM overview of sample A2248 6° viewed down [1 1 0] zone axis (a) with isolating areas with dislocations (b). Change the threshold to create a binary image (c) and making a skeleton image of the dislocations (d).*

Using Matlab, the number of pixels representing dislocations in the stamp filtered image was counted.. For pixel size for each skeleton image, starting from the In_x_Ga_1-x_As MB/GaAs interface. The pixel calibration was applied to yield the dislocation length and then applied to Eq. S1 alongside A and t.

Figure S5 demonstrates the WBDF images under **g_002_** for A2192 0.2°. Using the **g_002_** beam condition and tilting above, see that dislocations labelled 1,4,5 and 6 light up in the WBDF image as seen in Fig. S5b. However, we see that dislocations labelled 2, 3 and 7 do not. The opposite effect is seen when tilting below as seen in Fig. S5c. This can be an indication of dislocations which are asymmetric.


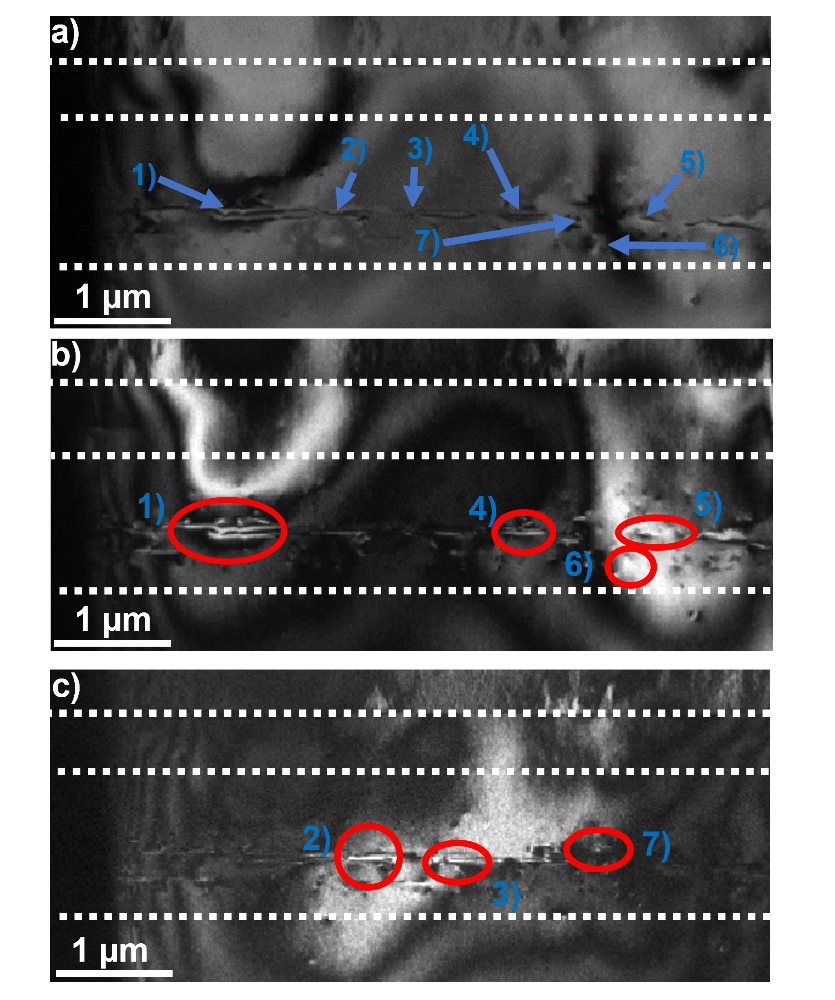


***Figure S5*** *Bright field TEM image (a) of A2192 0.2° and dark field image with* ***g_002_*** *upper band (b) and* ***g_002_*** *lower band (c). All images viewed down [1 1 0] zone axis.*

We now discuss in more detail the technique used to count dislocations. We started by taking an ADF- STEM of the MB as shown in Fig S6. Based on literature, dislocations with diagonal components (green line) were assigned as TD and those with straight line (blue line) or head (red circle) assigned as MD. The distance between In_x_Ga_1-x_As MB/ GaAs interface and the dislocation was measured (black arrow). For diagonal components, the measured distances were taken from the interface to the middle point of the dislocation and for the MD circled in red, the measured distance was from the interface to the centre spot of the circle.


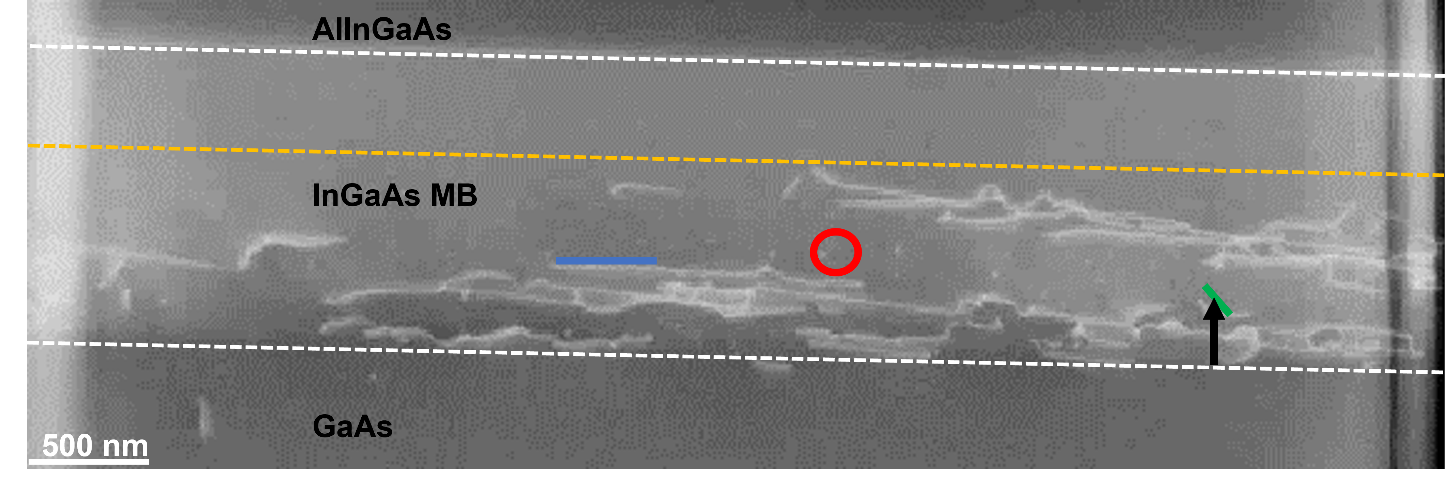


***Figure S6*** *Example ADF-STEM image used to analyse dislocation distribution in A2248 6°. The bottom and top white dotted line represents InGaAs MB/GaAs interface and AlInGaAs/InGaAs MB interface respectively with the orange line representing t_0_. Red circle and blue line indicate assigned example MD and green diagonal line indicates example TD. Distance of dislocation from In_x_Ga_1-x_As MB/GaAs Interface denoted with black arrow. Image is viewed down [1 1 0].*

| Sample | Total number of dislocations datapoints | Number of MD | Number of TD |
| --- | --- | --- | --- |
| A2192 0.2° | 64 | 47 | 17 |
| A2229 0.2° | 101 | 45 | 56 |
| A2248 6° | 177 | 114 | 63 |
| A2398 6° | 211 | 123 | 88 |

***Table S3*** *Summary of total number of dislocations identified with breakdown of MD and TD for Samples A2192 0.2°, A2229 0.2°, A2248 6° and A2398 6° used in Fig. 5.*

1. Strain mapping

The stages of GPA analysis will be discussed in detail. The first step is to crop edges of the image to reduce the image from a 2048×2048 pixel to a 1638×1638 pixel size image as seen in Fig. S7a. This was done to remove potential scanning artefacts at the edge of the images. We then apply a Fast Fourier transform (FFT) operation to image (Fig. S7b) and place a mask over the respective white spot to calculate either ε_yy_ or ε_xx_. For calculating ε_yy_ using Gatan, a mask is placed the location marked by the red circle in Fig. S7b while for ε_xx_ the mask is placed in the location of the green circle. The mask selected was a Gaussian Mask.

*
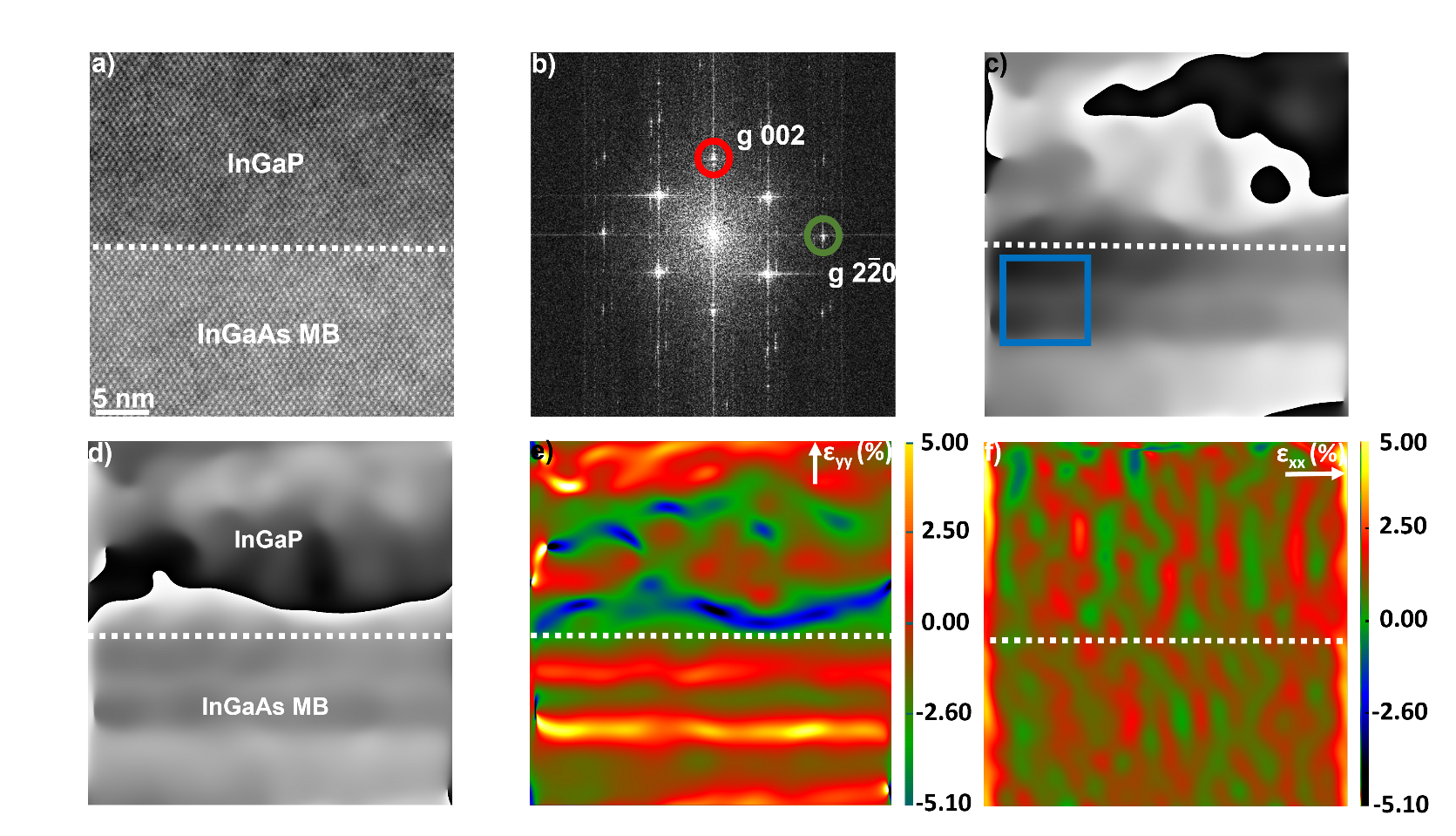
*

***Figure S7*** *Illustration of GPA strain map. HRSTEM image of area of interest a) and corresponding FFT image (b). With initial phase image (c) and final phase after tuned with respect to the reference region (d). Convert final phase image in GPA strain map (e). Red and green circle in (b) represent mask location for ε_yy_ and ε_xx_ respectively. Blue box in (c) represents reference region.*

A phase map for the FFT was collected as shown in Fig. S7c and tune with respect to a reference region. The accuracy of the phase depends on the quality of the image which can be greatly improved using a spherical aberration (Cs) corrected microscope. The size of the reference region as indicted by the red box is 410×410 pixels. Due to the nature of each phase map, the exact location of the reference region will differ slightly. We then tune twice over the reference region to obtain the phase map (Fig. S7d.) and covert to the GPA strain maps (Fig. S7e-f.).

To decide the mask size to use in GPA analysis, we tested various masks sizes measuring ε_yy_ for A2168 0.2° keeping other parameters identical. The sizes for the masks were 0.125**g**, 0.156**g** and 0.180**g** with the results outlined in Table S4. The smaller mask size is less accurate (-2.35±1.72% with theoretical strain of -0.02%) but comes with a lower uncertainty, compared to the medium 0.156**g** mask (-2.27±1.93%). This was also seen for comparing the small mask with the large 0.180**g** mask (-2.26±1.89%). Interestingly there was a 0.05% decrease in the uncertainty going from 0.156**g** mask to 0.180**g** mask. In this work, a mask size of 0.125g was used. Such a small mask size has the benefit of producing smoother results with a penalty in the spatial resolution. The compromise is that the smaller mask size is less accurate but comes with a lower uncertainty than other masks considered. Considering that the images were not corrected for (Cs) and that strain was measured over a wide area, the authors felt that the results from a mask that produced smoother results would be more suitable and give the best representation of the local strain. Another important consideration is that GPA involves selecting a reference region form the image and using the phase to calculate the strain.

| Mask size | A2168 0.2° ε_yy_ (%) |
| --- | --- |
| 0.125g | -2.35±1.72 |
| 0.156g | -2.27±1.93 |
| 0.180g | -2.26±1.89 |

*Table S4: ε_yy_ in A2168 0.2° as a function of mask size under identical conditions.*

*
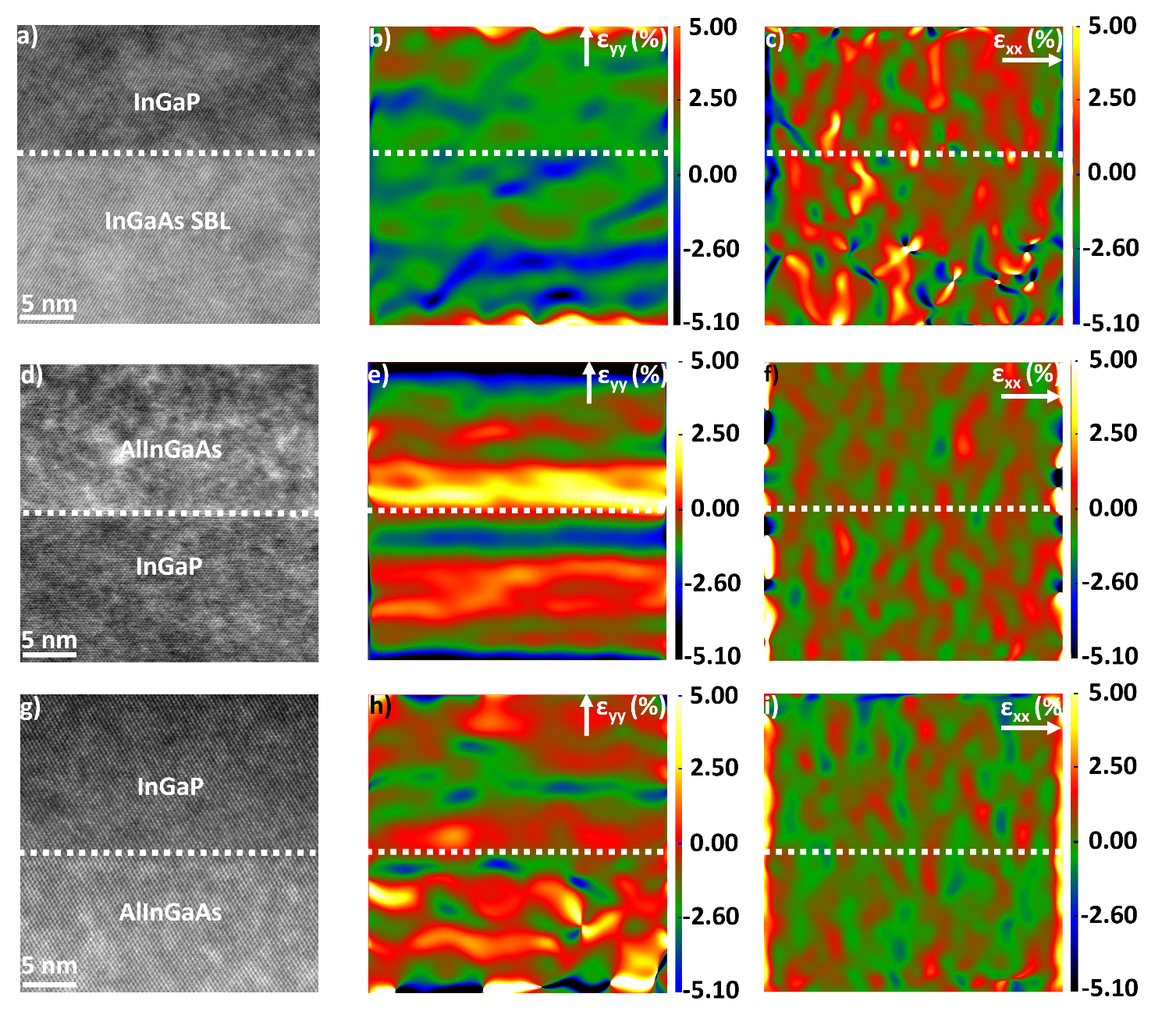
*

***Figure S8*** *HRSTEM of In_0.66_Ga_0.34_P/ In_0.13_Ga_0.87_As SBL interface in A2192 0.2° used for GPA (a) with ε_yy_ strain (b) and ε_xx_ (c) calculated. HRSTEM of Al_0.31_In_0.15_GaAs/ In_0.66_Ga_0.34_P interface in A2229 0.2° used for GPA (d) with ε_yy_ strain (e) and ε_xx_ (f) calculated. HRSTEM of In_0.62_Ga_0.38_P/Al_0.31_In_0.15_GaAs interface in A2248 6° used for GPA (g) with ε_yy_ strain (h) and ε_xx_ (i) calculated. All images viewed down [1 1 0] zone axis.*

As outlined in the main manuscript, non-Cs and Cs corrected STEM images of full metamorphic laser structure (A2398 6°) was measured to compare strain from GPA. STEM images such as Fig 9d were taken using a Nion UltraSTEM™ 100MC 'HERMES' at 100 kV fitted with a cold FEG. To minimise flyback error, rotation scan stacking reconstruction processing of 20 single images acquired 90° to each other was conducted.

***
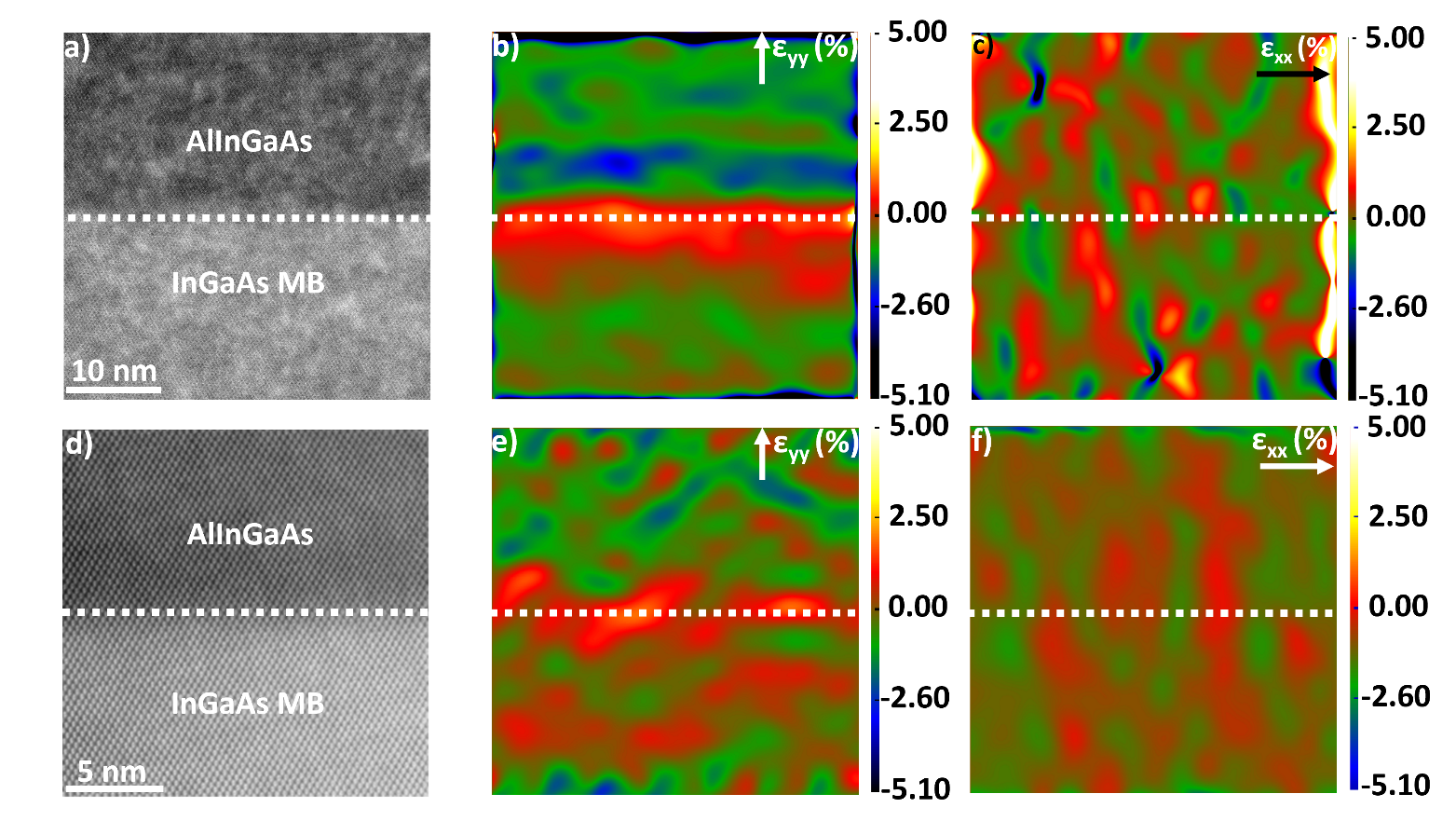
***

***Figure S9*** *HRSTEM of Al_0.31_In_0.15_GaAs /InGaAs MB interface in A2398 6° (non-Cs corrected) used for GPA (a) with ε_yy_ strain (b) and ε_xx_ (c) calculated. HRSTEM of Al_0.31_In_0.15_GaAs /InGaAs MB interface in A2398 6° (Cs corrected) used for GPA (d) with ε_yy_ strain (e) and ε_xx_ (f) calculated.*

Comparing the images (see Fig. S9.), we see that in there is build up of compressive ε_yy_ strain around the interface in the non-Cs corrected image while in the Cs corrected image, the ε_yy_ strain appears more homogenous across the sample. A potential reason for this could be attributed to differences in the thickness of the lamella. The lamella in the Cs corrected images were required to be thinner (<30nm) than those for non-Cs corrected (~60nm) and surface relaxation effects at thinner lamella thicknesses have been quoted to influence strain measurement. However in both the non-Cs and Cs corrected data, the AlInGaAs layer is under tensile strain in ε_yy_ within the same order of magnitude and in plane direction has minimum strain as shown in Table S5.

We now demonstrate how theoretical strain used compare the experimental results was calculated. Vegard’s Law is a method that is used to calculate the lattice parameter of ternary alloy such as In_x_Ga_1-x_As assuming that it can be thought of a mixture of InAs and GaAs. The general expression for the lattice parameter, a, of In_x_Ga_1-x_As is

$a\left( {In}_{x}{Ga}_{1-x}As \right)=x\left( a\left( InAs \right) \right)+\left( 1-x \right)\left( a\left( GaAs \right) \right)$ (Eq S2)

For example, as a(InAs)=0.60583nm and a(GaAs)=0.56355nm, a(In_0.18_Ga_0.82_As) is

$$a\left( {In}_{0.18}{Ga}_{0.82}As \right)=0.18\left( a\left( InAs \right) \right)+\left( 1-0.18 \right)\left( a\left( GaAs \right) \right)$$

$$=0.18\left( 0.60583nm \right)+\left( 1-0.18 \right)\left( 0.56355nm \right)$$

= 0.5763nm

Vegard’s law can also be extended to quaternary alloys such as AlInGaAs.

The strain from growing film layer on top of a substrate is

$\varepsilon= \frac{a_{f}-a_{s}}{a_{f}} \times100\%$ (Eq S3)

Where a_f_ and a_s_ are the lattice parameters of the film layer and substrate respectively. Suppose that we grow an In_0.66_Ga_0.34_P film on top of a In_0.12_Ga_0.88_As substrate. Using Vegard’s law, the lattice constant of In_0.66_Ga_0.34_P is 0. 5727nm and the lattice constant for In_0.13_Ga_0.87_As is 0.5706nm. Taking the values, the strain between In_0.66_Ga_0.34_P film and In_0.13_Ga_0.87_As substrate is

$$\varepsilon=\frac{0. 5727-0.5706}{0. 5727}\times100$$

=0.37%

| Sample | Layer | Reference Region | ε_xx_ | ε_yy_ | Theoretical ε |
| --- | --- | --- | --- | --- | --- |
| A2168 0.2° | In_0.66_Ga_0.34_P | InGaAs MB | -0.02±0.04 | -2.35±1.72 | -0.02 |
| A2168 6° | In_0.66_Ga_0.34_P | InGaAs MB | -0.25±0.04 | -0.16±0.42 | -0.02 |
| A2192 0.2° | In_0.66_Ga_0.34_P | In_0.13_Ga_0.87_As SBL | 0.16±0.07 | 0.90±0.31 | 0.37 |
| A2229 0.2° | Al_0.31_In_0.15_GaAs | In_0.66_Ga_0.34_P | 0.27±0.03 | 0.01±1.02 | 0.00 |
| A2248 6° | In_0.62_Ga_0.38_P | Al_0.31_In_0.15_GaAs | -0.05±0.07 | -1.04±0.75 | -0.06 |
| A2398 6° (non Cs corrected) | Al_0.31_In_0.15_GaAs | InGaAs MB | 0.02±0.03 | -1.03±0.38 | -0.02 |
| A2398 6° (Cs corrected) | Al_0.31_In_0.15_GaAs | InGaAs MB | -0.66±0.02 | -0.92±0.71 | -0.02 |

***Table S5*** *Tabulated summary strains in both ε_xx_ and ε_yy_ with theoretical strain direction layers noted with respect to outlined reference region for Samples A2168 0.2°, A2168 6°, A2192 0.2°, A2229 0.2°, A2248 6°* *and A2398 6°.*

It has been shown by Peters *et. al.* (see Peters JJP, Beanland R, Alexe M, *et. al.* (2015) Artefacts in geometric phase analysis of compound materials. Ultramicroscopy 157:91–97. https://doi.org/10.1016/j.ultramic.2015.05.020) that changing the **g**=**002** vector to **g**=**004** can help eliminate potential artefacts at the interface for these types of materials hence here we compare the strains under these conditions (see Fig S10-S12). Furthermore, we compare the output of the GPA results from two packages: 1) the Gatan script from Rouvière and 2)Strain ++.

We compare the output of the GPA results from the Gatan script with Strain ++ and changing g=**002** to g=**004** on samples A2248 6° and A2398 6°, as seen in Fig. S10-S12. In general, the values of the ε_xx_ and ε_yy_ fall within each other when using either Strain ++ or the Gatan script which shows the trends observe are valid (*i.e* for A2248 6° ε_xx_=-0.05±0.07% and .ε_yy_=-1.04±0.75% for the Gatan GPA script in comparison to ε_xx_= 0.05±0.03% and ε_yy_=-0.97±0.61% using Strain ++ with **g**=**002** and **g**=$\boldsymbol{2}\bar{\boldsymbol{2}}\boldsymbol{0}$). Looking at the strain profiles in Figure S13, we can see similarities in the strain profiles see which accounts for the agreement for the in both non-Cs and Cs corrected A2398 6° samples.

Table S6 summarises the findings of the different g vectors and software packages. From this we see that for both GPA packages, the ε_xx_ and ε_yy_ values lie within each other uncertainties after changing **g**=**002** to **g**=**004**. Furthermore, in some cases we observe that with the inclusion of uncertainty, value includes the theoretical prediction when **g**=**004** whilst using **g**=**002** was outside the predicated value (i.e for the Cs corrected A2398 6°, ε_yy_=-0.25±0.29% using **g**=**004**, ε_yy_=-0.97±0.71% with **g**=**002** and the theoretical strain was 0.06%).


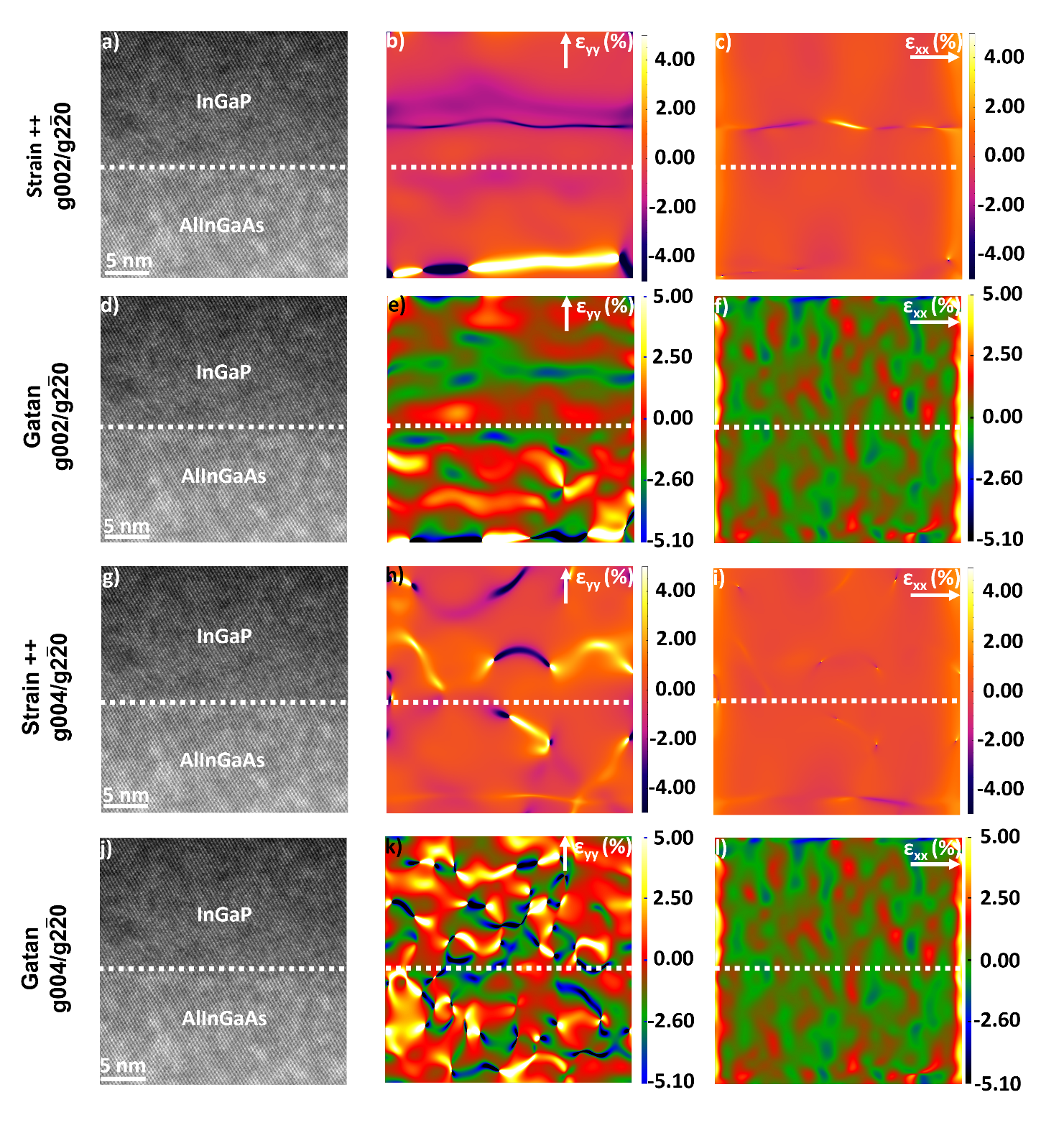


***Figure S10*** *Non-Cs corrected HRSTEM of AlInGaAs/ InGaAs MB interface in A2248 6° used for GPA (a) with ε_yy_ strain (b) and ε_xx_ (c) calculated from Strain ++ using* ***g****=****002*** *and* ***g***=$\text{2}\bar{\text{2}}\text{0}$ *. Non-Cs corrected HRSTEM of AlInGaAs/ InGaAs MB interface in A2248 6° used for GPA (d) with ε_yy_ strain (e) and ε_xx_ (f) calculated from the Gatan script using* ***g****=****002*** *and* ***g***=$\text{2}\bar{\text{2}}\text{0}$*. Non-Cs corrected HRSTEM of AlInGaAs/ InGaAs MB interface in A2248 6° used for GPA (g) with ε_yy_ strain (h) and ε_xx_ (j) calculated from Strain ++ using* ***g****=****004*** *and* ***g***=$\text{2}\bar{\text{2}}\text{0}$ *. Non-Cs corrected HRSTEM of AlInGaAs/ InGaAs MB interface in A2248 6° used for GPA (j) with ε_yy_ strain (k) and ε_xx_ (l) calculated from the Gatan script using* ***g****=****004*** *and* ***g***=$\text{2}\bar{\text{2}}\text{0}$*.*

*
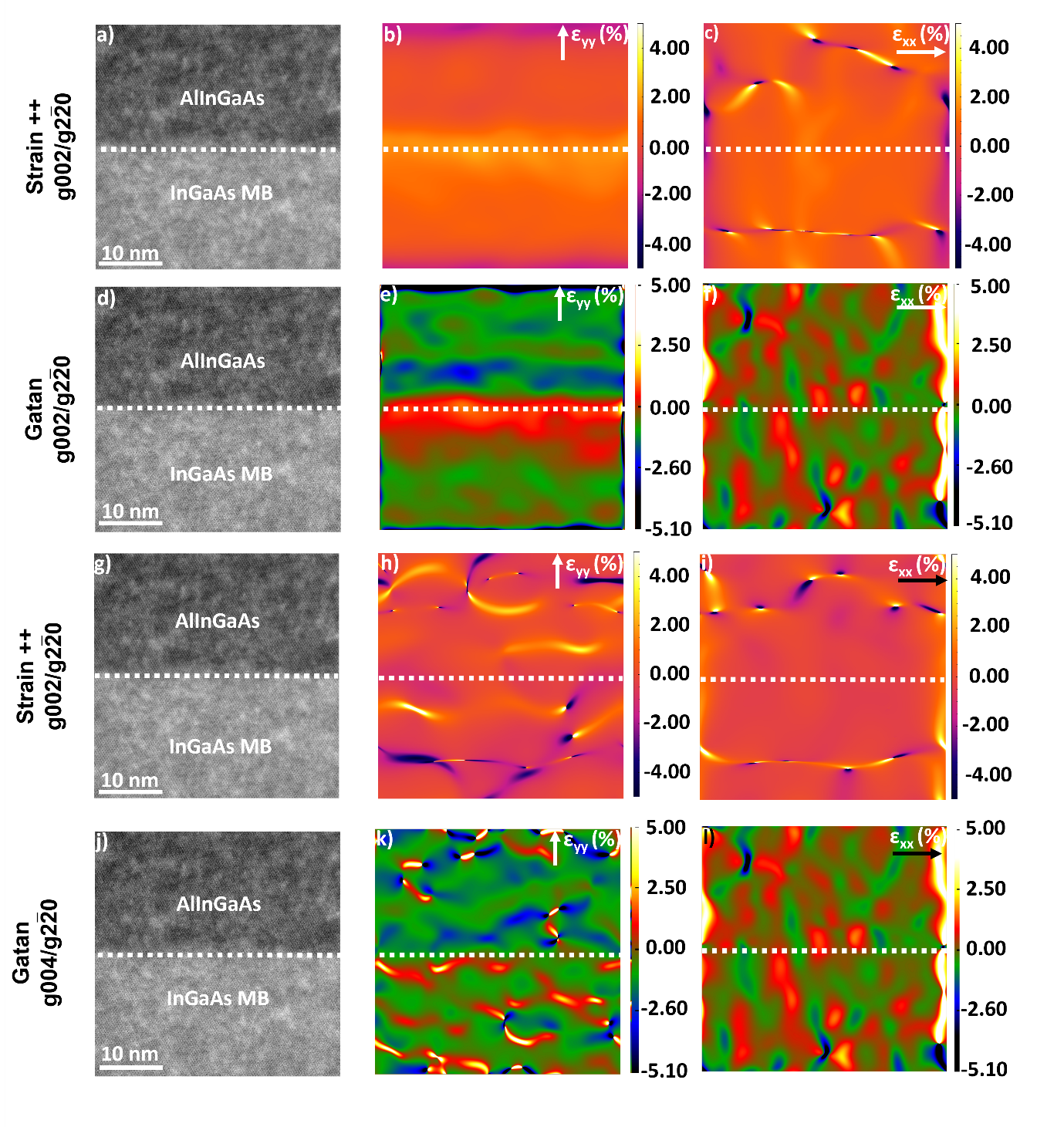
*

***Figure S11*** *Non-Cs corrected HRSTEM of AlInGaAs/ InGaAs MB interface in A2398 6° used for GPA (a) with ε_yy_ strain (b) and ε_xx_ (c) calculated from Strain ++ using* ***g****=****002*** *and* ***g***=$\text{2}\bar{\text{2}}\text{0}$*. Non-Cs corrected HRSTEM of AlInGaAs/ InGaAs MB interface in A2398 6° used for GPA (d) with ε_yy_ strain (e) and ε_xx_ (f) calculated from the Gatan script using* ***g****=****002*** *and* ***g***=$\text{2}\bar{\text{2}}\text{0}$*. Non-Cs corrected HRSTEM of AlInGaAs/ InGaAs MB interface in A2248 6° used for GPA (g) with ε_yy_ strain (h) and ε_xx_ (j) calculated from Strain ++ using* ***g****=****004*** *and* ***g***=$\text{2}\bar{\text{2}}\text{0}$ *. Non-Cs corrected HRSTEM of AlInGaAs/ InGaAs MB interface in A2398 6° used for GPA (j) with ε_yy_ strain (k) and ε_xx_ (l) calculated from the Gatan script using* ***g****=****004*** *and* ***g***=$\text{2}\bar{\text{2}}\text{0}$*.*


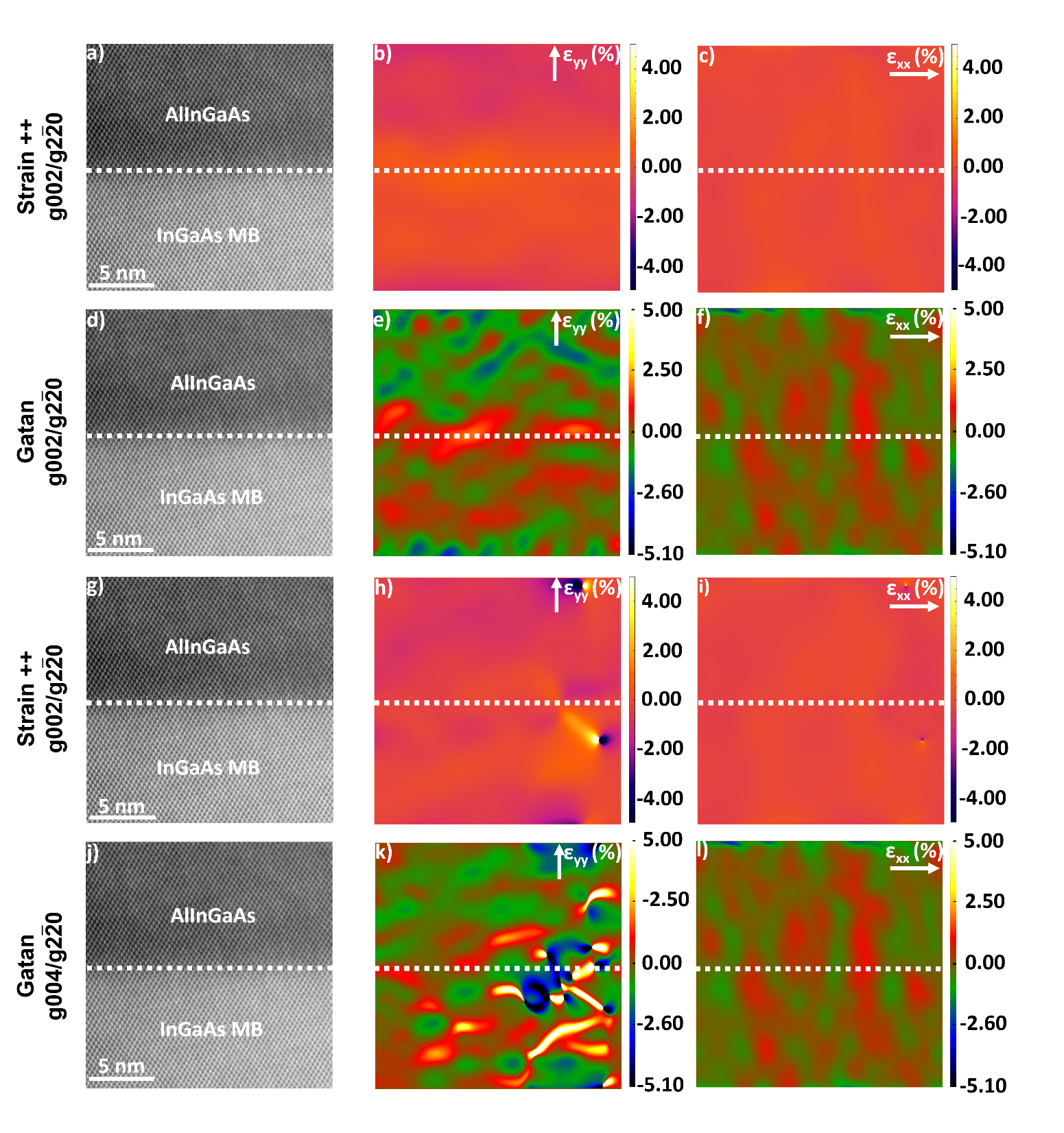


***Figure S12*** *Cs corrected HRSTEM of AlInGaAs/ InGaAs MB interface in A2398 6° used for GPA (a) with ε_yy_ strain (b) and ε_xx_ (c) calculated from Strain ++ using* ***g****=****002*** *and* ***g***=$\text{2}\bar{\text{2}}\text{0}$*. Cs corrected HRSTEM of AlInGaAs/ InGaAs MB interface in A2398 6° used for GPA (d) with ε_yy_ strain (e) and ε_xx_ (f) calculated from the Gatan script using* ***g****=****002*** *and* ***g***=$\text{2}\bar{\text{2}}\text{0}$*. Cs corrected HRSTEM of AlInGaAs/ InGaAs MB interface in A2248 6° used for GPA (g) with ε_yy_ strain (h) and ε_xx_ (j) calculated from Strain ++ using* ***g****=****004*** *and* ***g***=$\text{2}\bar{\text{2}}\text{0}$*. Cs corrected HRSTEM of AlInGaAs/ InGaAs MB interface in A2398 6° used for GPA (j) with ε_yy_ strain (k) and ε_xx_ (l) calculated from the Gatan script using* ***g****=****004*** *and* ***g***=$\text{2}\bar{\text{2}}\text{0}$*.*

Briefly state how the strain profile was measured from the GPA images. A line profile going up the sample as shown in Fig S13a,c. The width and location of the box were chosen such that no artefacts are included. The width of the line profile has a minimum width of 5nm to ensure a representative area. An overall strain for each layer was taken between the interface and the respective ends marked by the green lines. We should expect as the bottom layer (InGaAs MB for A2398 6°) is the reference layer, the bottom includes zero which in general was observed.


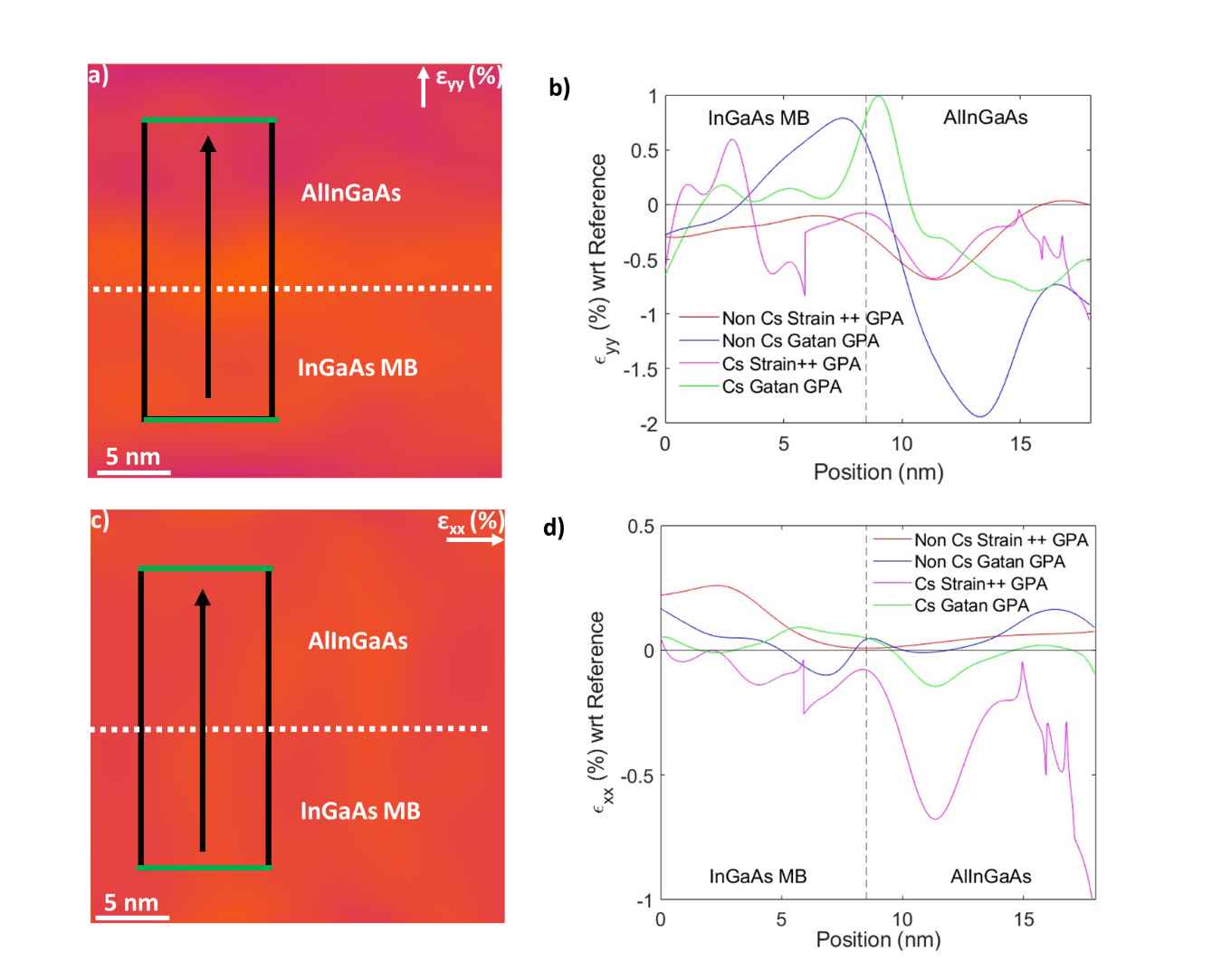


***Figure S13*** *ε_yy_ GPA image from Strain ++ of AlInGaAs/ InGaAs MB interface in A2398 6° (a) with black arrow and box denoting line direction and width of line profile used to obtain ε_yy_ profile (b). ε_xx_ GPA image from Strain ++ of AlInGaAs/ InGaAs MB interface in A2398 6° (c) with black arrow and box denoting line direction and width of line profile used to obtain ε_xx_ profile (d). All GPA shown here were conducted with* ***g****=****002*** *and* ***g***=$\text{2}\bar{\text{2}}\text{0}$*.*

| Sample | GPA used | g  vector | Cs  Corrected | Layer | ε_xx_ | ε_yy_ | Theoretical  ε |
| --- | --- | --- | --- | --- | --- | --- | --- |
| A2248 6° | S | **002** | No | In_0.62_Ga_0.38_P | 0.05±0.03 | -0.97±0.61 | -0.02 |
|  | G | **002** | No | In_0.62_Ga_0.38_P | -0.05±0.07 | -1.04±0.75 | -0.02 |
|  | S | **004** | No | In_0.62_Ga_0.38_P | 0.09±0.02 | 0.23±0.50 | -0.02 |
|  | G | **004** | No | In_0.62_Ga_0.38_P | -0.05±0.07 | 0.54±0.71 | -0.02 |
| A2398 6° | S | **002** | No | Al_0.31_In_0_._15_GaAs | 0.17±0.38 | -0.35±0.29 | -0.06 |
|  | G | **002** | No | Al_0.31_In_0_._15_GaAs | 0.02±0.03 | -1.03±0.38 | -0.06 |
|  | S | **002** | Yes | Al_0.31_In_0_._15_GaAs | 0.05±0.03 | -0.97±0.61 | -0.06 |
|  | G | **002** | Yes | Al_0.31_In_0_._15_GaAs | 0.03±0.03 | -0.92±0.71 | -0.06 |
|  | S | **004** | No | Al_0.31_In_0_._15_GaAs | 0.01±0.38 | 0.38±0.42 | -0.06 |
|  | G | **004** | No | Al_0.31_In_0_._15_GaAs | 0.02±0.03 | -1.07±0.39 | -0.06 |
|  | S | **004** | Yes | Al_0.31_In_0_._15_GaAs | -0.24±0.03 | -0.58±0.20 | -0.06 |
|  | G | **004** | Yes | Al_0.31_In_0_._15_GaAs | 0.17±0.38 | -0.25±0.29 | -0.06 |

***Table S6*** *Tabulated summary strains in both ε_xx_ and ε_yy_ with theoretical strain direction layers noted with respect to reference region for Samples A2248 6°* *and A2398 6°. S denotes Strain ++ and G is GPA calculated using the Gatan script. Reference regions are InGaP and InGaAs MB for A2248 6°* *and A2398 6° samples respectively.*
